# HNRNPK maintains epidermal progenitor function through transcription of proliferation genes and degrading differentiation promoting mRNAs

**DOI:** 10.1038/s41467-019-12238-x

**Published:** 2019-09-13

**Authors:** Jingting Li, Yifang Chen, Xiaojun Xu, Jackson Jones, Manisha Tiwari, Ji Ling, Ying Wang, Olivier Harismendy, George L. Sen

**Affiliations:** 1 Department of Dermatology, Department of Cellular and Molecular Medicine, UCSD Stem Cell Program, University of California, San Diego, La Jolla, CA 92093 USA; 2 Moores Cancer Center, University of California, San Diego, La Jolla, CA 92093 USA; 3 Department of Biomedical Informatics, University of California, San Diego, La Jolla, CA 92093 USA

**Keywords:** Genetics, Molecular biology, RNA metabolism, Transcription, Self-renewal

## Abstract

Maintenance of high-turnover tissues such as the epidermis requires a balance between stem cell proliferation and differentiation. The molecular mechanisms governing this process are an area of investigation. Here we show that HNRNPK, a multifunctional protein, is necessary to prevent premature differentiation and sustains the proliferative capacity of epidermal stem and progenitor cells. To prevent premature differentiation of progenitor cells, HNRNPK is necessary for DDX6 to bind a subset of mRNAs that code for transcription factors that promote differentiation. Upon binding, these mRNAs such as *GRHL3*, *KLF4*, and *ZNF750* are degraded through the mRNA degradation pathway, which prevents premature differentiation. To sustain the proliferative capacity of the epidermis, HNRNPK is necessary for RNA Polymerase II binding to proliferation/self-renewal genes such as *MYC*, *CYR61*, *FGFBP1*, *EGFR*, and cyclins to promote their expression. Our study establishes a prominent role for HNRNPK in maintaining adult tissue self-renewal through both transcriptional and post-transcriptional mechanisms.

## Abstract (web summary)

Maintenance of high turnover in tissues such as epidermis requires balance between proliferation and differentiation. Here the authors show that HNRNPK promotes RNA Polymerase II binding to proliferation and self-renewal genes as well as degradation of differentiation promoting mRNAs together with DDX6 in epidermis.

## Introduction

Mammalian epidermis is the outermost layer of the skin, which serves as the initial line of defense to protect our body from environmental and pathogenic factors. The deepest layer of the epidermis, known as the basal layer, contains the undifferentiated stem and progenitor cells. As the cells differentiate they exit out of the cell cycle and migrate upwards towards the surface of the skin and progressively form the more differentiated layers of the epidermis including the suprabasal and granular layers. Terminal differentiation occurs in the stratum corneum where these dead corneocytes eventually gets sloughed off the surface of the skin. Epidermal homeostasis is achieved by the proper balance between self-renewal and differentiation of stem and progenitor cells residing within the basal layer^1^. Perturbations in this delicate balance can lead to skin diseases which can impact up to 20% of the population^2^. A tremendous amount of effort has been focused on defining transcriptional mechanisms that regulate epidermal stem and progenitor cell self-renewal and differentiation. Work by our laboratory and others have shown that transcription and epigenetic factors such as p63, DNMT1, ACTL6A, YAP1, and EZH2 can actively promote epidermal stem and progenitor cell self-renewal while factors such as ZNF750, KLF4, GRHL3, JMJD3, and CEBP alpha/beta are necessary for differentiation^3–13^.

While transcriptional mechanisms that regulate epidermal self-renewal or differentiation have been well described as discussed above, it is not clear whether post-transcriptional mechanisms (non-miRNA) regulate this process. Recently, we found the DEAD-box RNA helicase, RCK/p54 (DDX6) to be necessary for maintaining epidermal stem and progenitor cell function through both the mRNA translation and degradation pathways^14,15^. DDX6 promotes self-renewal and proliferation while actively suppressing premature differentiation through two distinct mechanisms. First, DDX6 promotes the translation of self-renewal (*EZH2*, *ACTL6A*) and proliferation (*CDK1*, *CDK2*) transcripts to maintain epidermal self-renewal. The RNA binding protein, YBX1 recruits DDX6 and EIF4E to these self-renewal/proliferation mRNAs through the stem loop structures found in their 3’UTRs. EIF4E is then required for the initiation of translation of these transcripts while DDX6 is necessary for loading of the mRNAs to polysomes. Second, DDX6 binds to mRNAs coding for potent differentiation promoting transcription factors such as *KLF4* to promote its degradation in progenitor cells to prevent premature differentiation. DDX6 promotes the degradation of these transcripts by associating with key mediators of the mRNA degradation pathway including EDC3^14–16^. Currently, it is unclear how DDX6 targets these mRNAs for degradation since YBX1 recruits DDX6 to self-renewal/proliferation transcripts but not differentiation mRNAs such as *KLF4*^14,15^.

In an attempt to identify proteins that recruit DDX6 to differentiation promoting mRNAs for degradation, we perform a small RNA interference (RNAi) screen targeting RNA binding proteins that we previously identified by mass spectrometry to associate with DDX6^14^. Through this screen we find heterogeneous nuclear ribonucleoprotein K (HNRNPK) to have a prominent role in epidermal progenitor cell maintenance. HNRNPK was initially discovered as a component of hnRNP complexes of which there were originally 20 proteins identified and named A1 to U^17^. It contains three K homologue (KH) domains that can bind RNA or DNA. HNRNPK has been prominently studied in cancer and has been described as both an oncogene and tumor suppressor. HNRNPK has been found to be overexpressed in a variety of tumors with its oncogenic functions attributed to regulation of EIF4E, C-SRC, and C-MYC^18–23^. In epithelial tumors, HNRNPK interacts with Keratin 17 to promote tumor growth through inflammatory responses mediated through the CXCR3 and AIRE pathways^24,25^. Its tumor suppressive roles were uncovered in haploinsufficient mice which resulted in hematopoietic neoplasms^26^. HNRNPK has been implicated in all aspects of gene regulation including both transcriptional and post-transcriptional mechanisms. This includes regulation of transcription, mRNA stability, splicing, export, and translation^23^. While there is a wide array of gene regulatory functions attributed to HNRNPK, it is not clear whether HNRNPK performs more than one of these functions at a time since previous studies have only focused on one aspect of its gene regulatory functions. It is also unclear whether HNRNPK has any role in adult stem cell or tissue renewal since knockout mice are embryonic lethal^26^. This brings up an interesting and exciting question of whether cell fate choices such as the decision for a stem cell to renew or differentiate can be controlled by both transcriptional and post-transcriptional mechanisms through a single factor.

Here, we show that HNRNPK prevents premature differentiation of human epidermal progenitor cells by degrading mRNAs coding for differentiation promoting transcription factors through the DDX6 pathway. HNRNPK is also necessary to sustain the proliferative capacity of progenitor cells through transcriptional regulation of critical proliferation genes. These findings highlight the importance of HNRNPK in regulating adult tissue homeostasis by utilizing both transcriptional and post-transcriptional mechanisms.

## Results

### HNRNPK promotes proliferation and prevents differentiation

**Fig. 1:**
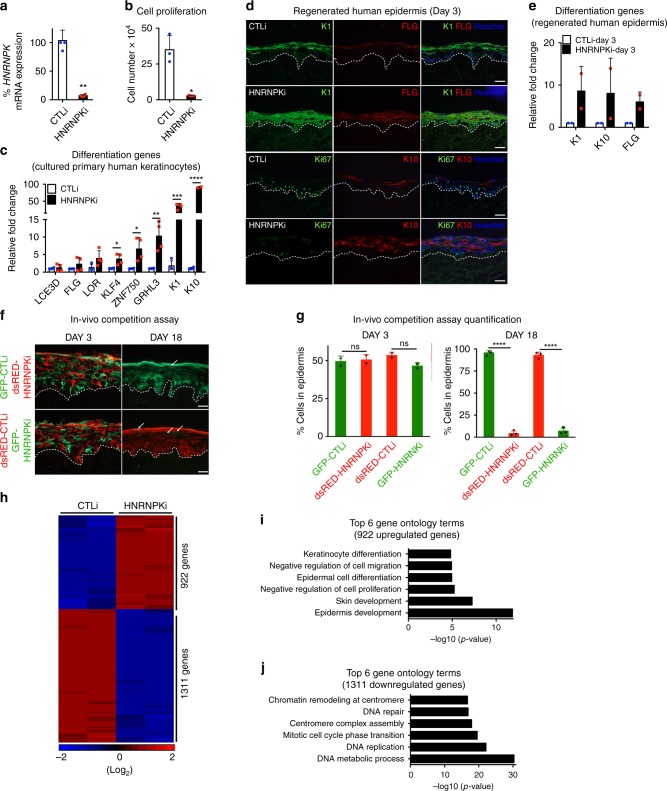
HNRNPK is necessary to sustain epidermal progenitor cell function. **a** Epidermal progenitor cells were knocked down with control (CTLi) or HNRNPK (HNRNPKi) siRNAs and the remaining *HNRNPK* mRNA levels were measured by RT-QPCR. QPCR results were normalized to *L32* levels. *n* = 3 independent experiments **b** CTLi and HNRNPKi cells were seeded at 100,000 and counted 4 days later. **c** RT-QPCR for epidermal differentiation gene expression in CTLi and HNRNPKi cells. QPCR results were normalized to *L32* levels. *n* = 4. **d** Human epidermis was regenerated using three-dimensional organotypic cultures with CTLi or HNRNPKi cells. Expression of differentiation markers (K1, K10, and FLG) and proliferation index (Ki67) were characterized by immunostaining at day 3. White scale bar = 20 μm, *n* = 2. **e** RT-QPCR for epidermal differentiation gene expression in CTLi and HNRNPKi regenerated human epidermis. *n* = 2. **f** The in-vivo progenitor cell competition assay was performed by mixing an equal number of GFP or dsRED labeled CTLi or HNRNPKi cells and used to regenerate human epidermis on immune-compromised mice. Skin grafts were harvested at days 3 and 18 post-grafting. shRNA knockdown of HNRNPK in-vivo was mediated through retroviral gene transfer to obtain stable gene suppression. The dashed white lines denote the basement membrane zone. White arrows mark remaining HNRNPKi cells in the epidermis. *n* = 3 animals grafted per timepoint per group. White scale bar = 20 μm. **g** Quantitation of the percentage of GFP and dsRED cells remaining in the epidermis at days 3 and 18 using ImageJ. **h** RNA-Seq analysis of CTLi and HNRNPKi cells. Cells were cultured for 7 days and subjected to RNA-Seq. Heatmap of the genes that change upon HNRNPK depletion are shown in red (induced) and blue (downregulated) on a log2 scale. RNA-Seq was performed in biological replicates. **i** Top 6 gene ontology terms for the genes upregulated in HNRNPKi cells using Enrichr. **j** Gene ontology terms for the downregulated genes in HNRNPKi cells using Enrichr. *n* = 3 independent experiments performed in Fig. 1 unless otherwise indicated. Mean values are shown with error bars = SD. *p < 0.05, **p < 0.01, ***p < 0.001, ****p < 0.0001: **a**, **b**, **c**, **e**, **g**: *t*-test (two sided)

To identify proteins that can potentially recruit DDX6 to degrade mRNAs that code for differentiation inducing transcription factors such as KLF4, a small RNA interference (RNAi) screen was performed. We previously showed that knockdown of DDX6 in epidermal progenitor cells led to increased *KLF4* mRNA stability and expression^14^. Thus, we knocked down all seven of the RNA binding proteins that we previously found by mass spectrometry to associate with DDX6 to determine if they have similar impacts on *KLF4* expression (Supplementary Fig. 1a)^14^. Of the seven genes, knockdown of HNRNPK resulted in an increase of *KLF4* gene expression levels (Supplementary Fig. 1b). RNAi knockdown of HNRNPK in primary human keratinocytes using two distinct sequences [HNRNPKi and HNRNPK-Bi] inhibited proliferation by more than 80% as compared to knockdown controls (CTLi) (Fig. 1a, b and Supplementary Fig. 1c, d). There was also an increase in apoptotic cells upon HNRNPK knockdown although it was not statistically significant (Supplementary Fig. 1e). HNRNPK knockdown cells also prematurely differentiated with increased levels of differentiation specific genes many of which have been implicated in skin diseases including *KRT1*, *KRT10*, *FLG*, and *LOR* (Fig. 1c and Supplementary Fig. 1f)^27–30^. Notably, the mRNAs levels (*KLF4*, *ZNF750*, and *GRHL3*) for genes that code for differentiation promoting transcription factors were also increased (Fig. 1c and Supplementary Fig. 1f).

To determine the impacts of HNRNPK loss in a 3D tissue setting which allows faithful representation of the stratification and gene expression program of human epidermis, CTLi and HNRNPKi cells were used to regenerate human epidermal tissue^7,31,32^. Epidermal tissue was harvested at an early timepoint to determine if HNRNPK depletion altered the kinetics of differentiation. Keratin 1 (K1), a differentiation specific cytoskeletal protein, which is normally only expressed beginning in the suprabasal layer was robustly expressed in the basal layer of HNRNPKi tissue suggesting that the stem cell compartment had prematurely differentiated (Fig. 1d, e). Similarly, keratin 10 (K10) and Filaggrin (FLG) were expressed robustly in HNRNPKi tissue whereas expression of these proteins was just starting to appear in control tissue (Fig. 1d, e). Coinciding with the premature differentiation of the stem/progenitor cell compartment in HNRNPKi tissue, there was also a loss of the proliferative capacity of the basal layer (Fig. 1d).

### HNRNPK maintains epidermal self-renewal in-vivo

To determine whether HNRNPK is acting through non-cell autonomous or cell autonomous mechanisms, as well as whether HNRNPK is necessary for progenitor cell function in-vivo, we used the competition assay we previously developed^11,14,33,34^. To do this, GFP expressing keratinocytes were knocked down with control (CTLi) shRNAs and dsRED cells were knocked down with HNRNPK shRNAs. These cells were mixed at a 1:1 ratio. All genetic modifications such as gene overexpression or knockdown were mediated through retroviral infections and thus are stable in-vivo^11,14,33,34^. To rule out impacts from the fluorescent proteins, dsRED expressing CTLi cells were also mixed with GFP expressing HNRNPKi cells at equal ratios. These different cell mixtures were used to regenerate human epidermis on immune deficient mice. Initially (Day 3), epidermis generated from GFP-CTLi + dsRED-HNRNPKi cells or GFP-HNRNPKi + dsRED-CTLi cells showed an equal percentage of contribution to the tissue (Fig. 1f, g). In contrast, by 18 days post-grafting, epidermis regenerated from GFP-CTLi mixed with dsRED-HNRNPKi cells were mainly composed of control GFP cells (Fig. 1f, g). Epidermis derived from dsRED-CTLi mixed with GFP-HNRNPKi cells also led to a huge depletion of the GFP expressing HNRNPK knockdown cells. The few remaining GFP-HNRNPKi cells were all found in the upper differentiated layers of the epidermis (Fig. 1f: white arrowheads). These remaining cells will likely be sloughed off the surface of the skin as they terminally differentiate.

### HNRNPK suppresses differentiation and promotes proliferation

RNA-seq analysis was performed on control and HNRNPKi cells to understand the gene expression program that HNRNPK controls. Nine hundred and twenty-two genes (≥2 fold change and *p* ≤ 0.05, one way Anova) were upregulated in HNRNPKi cells which were enriched in gene ontology (GO) terms such as keratinocyte differentiation, negative regulation of cell proliferation and epidermis development (Fig. 1h, i and Supplementary Data 1). 1311 genes were downregulated with GO terms including mitotic cell cycle phase transition and DNA replication indicating that HNRNPK is necessary for the proliferative capacity of the epidermal cells (Fig. 1h, j and Supplementary Data 1). To determine the extent to which depletion of HNRNPK resembles the differentiation program, the HNRNPK signature was compared to a previously generated RNA-seq data set of genes that changed during calcium mediated epidermal differentiation^35^. Nine hundred and fourteen genes were found in the overlap with the vast majority of the genes regulated in the same direction as the differentiation signature (Supplementary Fig. 1g). The overlapped upregulated genes were enriched in GO terms such as keratinocyte differentiation while downregulated genes were enriched in mitotic cell cycle (Supplementary Fig. 1h, i).

### HNRNPK degrades differentiation promoting mRNAs

**Fig. 2:**
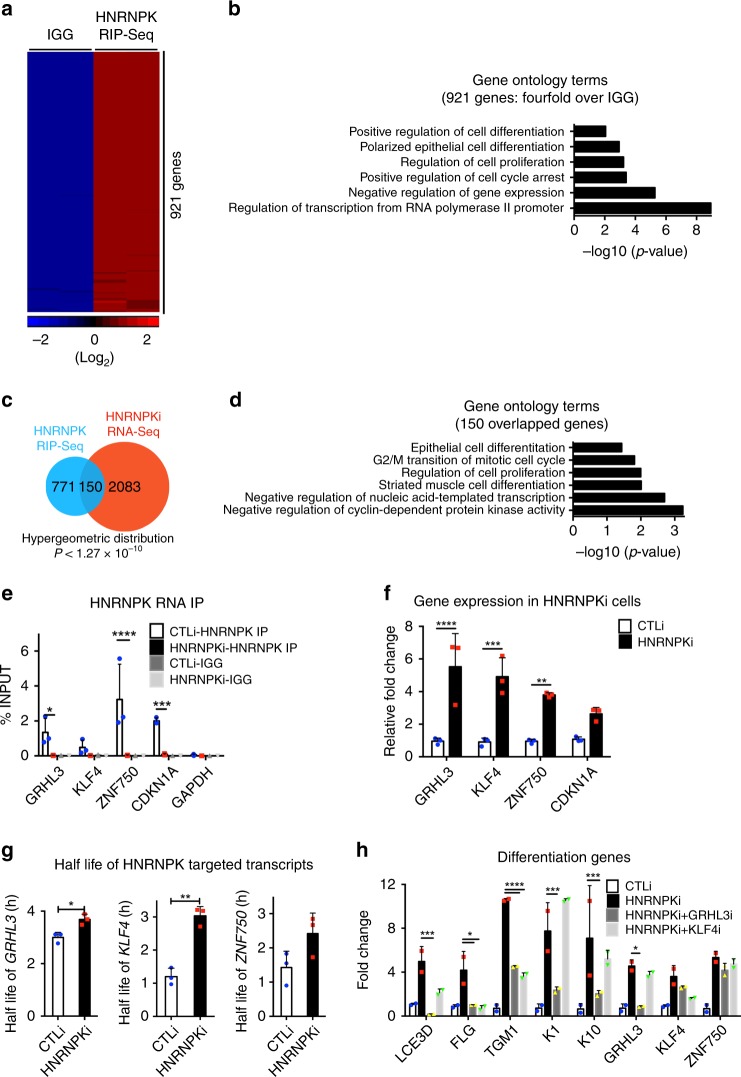
HNRNPK binds and degrades mRNAs coding for differentiation promoting transcription factors to prevent premature differentiation. **a** Profiling of HNRNPK bound transcripts by RNA immunoprecipitation (RNA IP) coupled with deep sequencing (RIP-Seq). Heatmap of 921 genes bound to HNRNPK defined by 4-fold enrichment over IGG and *p* < 0.05, one way Anova. *n* = 2 independent experiments. **b** Gene ontology terms of the 921 genes bound by HNRNPK using Enrichr. **c** Venn diagram of overlapped genes between HNRNPKi RNA-Seq and HNRNPK RIP-Seq datasets. **d** Gene ontology terms of the 150 overlapped genes. **e** RNA IP was performed in CTLi and HNRNPKi cells using an HNRNPK antibody. RT-QPCR was used to determine the levels of binding between HNRNPK and *GRHL3*, *KLF4*, *ZNF750, GAPDH*, or *CDKN1A* mRNAs in CTLi and HNRNPKi cells. IGG IPs in CTLi and HNRNPKi cells were used as specificity controls. Binding was calculated as a percent of input. **f** RT-QPCR for changes in the levels of *GRHL3*, *KLF4*, *ZNF750*, and *CDKN1A* mRNA expression in HNRNPKi cells. QPCR results were normalized to *L32* levels. **g** CTLi and HNRNPKi cells were treated with actinomycin D to determine the half-lives of the differentiation associated transcripts. RT-QPCR was used to measure the levels of the transcripts. **h** Double knockdown of HNRNPK with GRHL3 or KLF4 was performed and differentiation markers were evaluated by RT-QPCR (*n* = 2). *n* = 3 independent experiments performed in Fig. 2 unless otherwise indicated. Mean values are shown with error bars = SD. *p < 0.05, ** *p* < 0.01, ***p < 0.001, ****p < 0.0001 (2 way ANOVA followed by Tukey’s multiple comparison test for **h**, **e**. *T*-test for **f**, **g**). Overlap significance in Venn diagrams was determined using hypergeometric distribution *p*-values (**c**)

Since HNRNPK has been shown to bind RNA to modulate post-transcriptional gene expression, we performed RNA immunoprecipitations (RIP) under native conditions using a HNRNPK antibody or control IgG on epidermal cell lysates to determine all the transcripts that HNRNPK binds. RNA was purified from the immunoprecipitates and subjected to next generation RNA sequencing (RIP-Seq)^36,37^. HNRNPK bound to 921 genes (≥4 fold increase over IgG and *p* ≤ 0.05, one way Anova) which were enriched for GO terms including positive regulation of cell differentiation, polarized epithelial cell differentiation, and regulation of cell proliferation (Fig. 2a, b and Supplementary Data 2). There was also a tight correlation between the replicate HNRNPK RIP-Seq data sets (Supplementary Fig. 1j). To assess whether HNRNPK bound transcripts also changed in expression levels upon HNRNPK depletion, the HNRNPK RIP-Seq dataset was overlapped with the HNRNPK knockdown data. Interestingly, 16% (150/921) of the HNRNPK bound genes were differentially regulated upon HNRNPK knockdown which were enriched for GO terms such as epithelial cell differentiation, regulation of cell proliferation, and negative regulation of cyclin-dependent protein kinase activity (Fig. 2c, d). These results suggest that HNRNPK may regulate epidermal growth and differentiation at the post-transcriptional level. Among the genes involved in regulating epithelial cell differentiation and growth that is both bound by HNRNPK and found to be increased in mRNA levels upon knockdown were transcripts coding for differentiation promoting transcription factors including *KLF4*, *ZNF750*, and *GRHL3,* as well as the cell cycle inhibitor P21 (*CDKN1A)* (Figs. 1h, 2a, c).

To validate the RIP-Seq results, RIP was performed in control and HNRNPK depleted cells using a HNRNPK antibody or IgG. *KLF4*, *ZNF750*, *CDKN1A*, and *GRHL3* mRNAs were found to robustly associate with HNRNPK in control but not in HNRNPKi cells (Fig. 2e, Supplementary Fig. 2a). The transcripts were specifically bound to HNRNPK since binding depended on the presence of HNRNPK in the cells and did not bind transcripts such as *GAPDH* (Fig. 2e). No binding was detected in the IgG pulldown samples (Fig. 2e, Supplementary Fig. 2a). Since knockdown of HNRNPK led to increases in the mRNA levels of these HNRNPK bound genes, it suggests that HNRNPK may normally be targeting these transcripts for degradation in progenitor cells to prevent premature differentiation and premature cell cycle exit (Fig. 2f). To test this, control and HNRNPKi cells were treated with actinomycin D to determine the half-lives of the mRNAs. Loss of HNRNPK significantly increased the mRNA stability/half-lives of *GRHL3*, *KLF4*, and *CDKN1A* (Fig. 2g, Supplementary Fig. 2b). While not statistically significant, HNRNPK depletion also led to the increased half-life of *ZNF750* (Fig. 2g). These results suggest that HNRNPK binds and degrades these transcripts in progenitor cells to prevent premature differentiation.

To determine if HNRNPK may be regulating growth and differentiation through these bound genes, we overlapped our published gene expression signatures of KLF4 and ZNF750 knockdown in differentiated keratinocytes with our HNRNPK gene expression profile^7^. Since we have shown that KLF4 and ZNF750 are required for epidermal differentiation we would expect the knockdown expression profiles to be counter correlated. One hundred and sixty-six genes were upregulated in HNRNPK knockdown and downregulated in KLF4i cells which were enriched for GO terms such as epidermal cell differentiation and skin development (Supplementary Fig. 3a, b). Three hundred and sixty-six genes were downregulated in HNRNPKi and upregulated in KLF4i cells which were enriched in GO terms such as positive regulation of cell cycle (Supplementary Fig. 3a, c). Similar results were obtained with the ZNF750 overlap suggesting that these factors may mediate HNRNPK’s impact on growth and differentiation (Supplementary Fig. 3d-f).

To directly determine if the impacts of HNRNPK on epidermal growth and differentiation is mediated through the increased expression of *GRHL3*, *KLF4*, or *CDKN1A*, HNRNPK and each of the genes were simultaneously knocked down and compared to control and HNRNPKi cells (Fig. 2h). The levels of differentiation induced genes *LCE3D*, *FLG*, *TGM1*, and *K10* were increased in HNRNPKi cells but were restored similar to control levels in HNRNPK + GRHL3 or HNRNPK + KLF4 double knockdown cells (Fig. 2h). Simultaneous HNRNPK and CDKN1A knockdown had no impact on the premature differentiation phenotype (Supplementary Fig. 2c). These results suggest that HNRNPK prevents premature differentiation of epidermal progenitor cells by binding to and degrading *GRHL3* and *KLF4* transcripts.

To determine if HNRNPK promotes epidermal growth through suppression of these transcripts, double knockdown experiments were performed and cell number counted 5 days after normalization of the cell number. Again, HNRNPKi cells exhibited dramatic inhibition of proliferation with loss of expression of key proliferation genes such as *CCNA2*, *CDK4*, and *MKI67*. Double knockdown of HNRNPK with GRHL3, KLF4 or CDKN1A resulted in a similar loss of proliferation as HNRNPK knockdown alone and did not restore the proliferation back to control levels (Supplementary Fig. 2d-g). It is intriguing to note that HNRNPK + CDKN1A double knockdown did not correct the proliferation defect even though CDKN1A is a major cell cycle inhibitor that upon knockdown by itself led to ~4 increase in cell numbers as compared to control cells (Supplementary Fig. 2d).

### HNRNPK is necessary for DDX6 to bind differentiation mRNAs

**Fig. 3:**
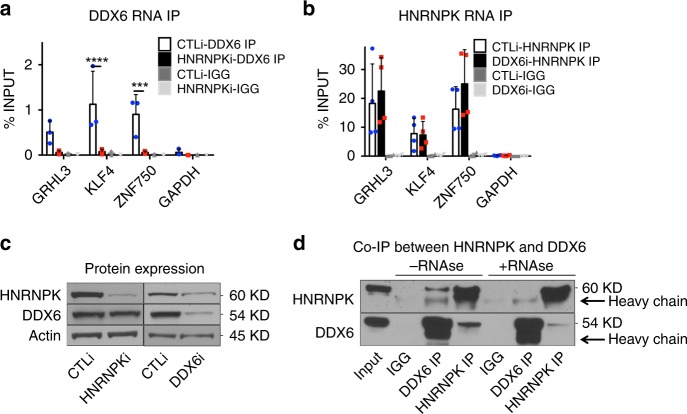
HNRNPK is necessary for DDX6 to bind differentiation associated mRNAs. **a** RNA IP was performed in CTLi and HNRNPKi cells using a DDX6 antibody. RT-QPCR was used to determine the levels of binding between DDX6 and differentiation associated mRNAs in the presence or absence of HNRNPK. IGG IPs in CTLi and HNRNPKi cells were used as specificity controls. Binding was calculated as a percent of input. **b** RNA IP was performed in CTLi and DDX6i cells using a HNRNPK antibody. RT-QPCR was used to determine the levels of binding between HNRNPK and differentiation associated mRNAs in the presence or absence of DDX6. IGG IPs in CTLi and DDX6i cells were used as specificity controls. *n* = 4. **c** Western blot analysis of HNRNPK and DDX6 protein levels upon HNRNPK or DDX6 knockdown. **d** Immunoprecipitations (IPs) were performed using either an HNRNPK or DDX6 antibody or IGG and Western blotted for HNRNPK or DDX6 protein expression. IPs were performed +/− RNase A. Five percent of the cell lysate was used as input. Representative blots are shown. *n* = 3 independent experiments performed for Fig. 3 unless otherwise indicated. All error bars = SD. ****p < 0.0001, ***p < 0.001 (2 way ANOVA followed by Tukey’s multiple comparison test for **a**, **b**)

We previously demonstrated that DDX6 degrades *KLF4* transcripts however it was unclear how DDX6 was targeted to the mRNA^14^. To determine whether HNRNPK recruits the targeted mRNAs to DDX6 or vice versa, RIP experiments were performed. RIP was performed using a DDX6 antibody or IgG in control and HNRNPKi cells. DDX6 was able to bind to *KLF4*, *ZNF750*, and *GRHL3* transcripts in control but not HNRNPK depleted cells suggesting that HNRNPK is necessary for DDX6 to associate with its target mRNAs (Fig. 3a). HNRNPK depletion had no impacts on DDX6 protein levels suggesting that the loss of DDX6 binding to transcripts is not due to an absence of its protein (Fig. 3c). In the reverse experiment, the mRNAs associated with HNRNPK were pulled down in both control and DDX6i cells. Despite, the slight decrease of HNRNPK protein levels upon DDX6 knockdown, HNRNPK was still able to bind *KLF4*, *ZNF750*, and *GRHL3* transcripts robustly in the absence of DDX6 (Fig. 3b, c). These results suggest that HNRNPK recruits the mRNAs to DDX6, which can then degrade the transcripts. HNRNPK may potentially associate with DDX6 directly or through the targeted transcripts. To test this, co-immunoprecipitations (Co-IPs) of HNRNPK and DDX6 were performed in the presence or absence of RNase A. In the absence of RNase A, DDX6 and HNRNPK could associate with each other (Fig. 3d). However upon RNase A addition, the association was diminished suggesting that the interaction was mediated primarily through the binding of RNA (Fig. 3d). To gain insight into how much of the HNRNPKi gene expression signature and thus regulation of epidermal growth and differentiation is through the DDX6 pathway, we compared our previously published DDX6 knockdown mRNA expression signature with it^14^. Sixty six percent (336/521) of the DDX6 signature overlapped with HNRNPK which included significant genes involved in epidermal development and cell cycle (Supplementary Fig. 3g, h). This suggests that a majority of the DDX6 function is mediated through HNRNPK. However, the DDX6 signature only accounts for ~15% (336/2233) of the HNRNPK signature suggesting that HNRNPK can potentially mediate its effects on epidermal growth and differentiation through DDX6 independent mechanisms.

### Genome-wide binding sites of HNRNPK

**Fig. 4:**
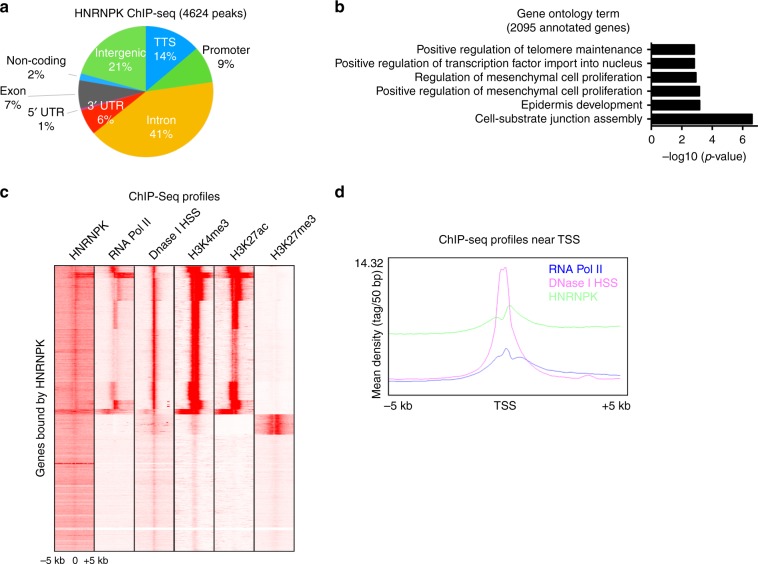
ChIP-Seq analysis of HNRNPK genome-wide binding sites. **a** The 4,624 HNRNPK significantly bound peaks were mapped back to various regions of the genome. The percentage of the HNRNPK peaks found within each region of the genome is shown. **b** The 4,624 HNRNPK peaks were mapped back to its nearest genes (2095) and gene ontology analysis performed to determine enrichment for gene function. **c** Heatmap of genes bound by HNRNPK centered on the TSS (±5 kb region) for RNA Pol II, DNase-I-hypersensitive sites (DNase I HSS), H3K4me3, H3K27ac, and H3K27me3. **d** The distribution of DNase I HSS, RNA Pol II, and HNRNPK binding from ±5 kb to TSS of genes. The *y*-axis denotes signal strength (mean density of the reads) and x-axis shows position from the TSS

HNRNPK has been reported to be a multi-faceted protein which can also bind to DNA and regulate gene expression through transcriptional mechanisms. To explore this possibility, chromatin immunoprecipitation (ChIP) followed by deep sequencing (ChIP-Seq) was performed using a HNRNPK antibody on primary human keratinocytes. MACS2 was used to call 4624 peaks enriched with HNRNPK binding (Supplementary Data 3a)^38,39^. A majority (68%) of the binding was found within genes which includes the 5’ UTR, intron, exon, transcriptional termination site/TTS, and 3’ UTR (Fig. 4a). The rest of the peaks were within intergenic, promoter, or non-coding regions (Fig. 4a). The peaks mapped back to 2,095 genes which were enriched for GO terms such as positive regulation of telomere maintenance, epidermis development, and positive regulation of mesenchymal cell proliferation (Fig. 4b and Supplementary Data 3a). To gain a better understanding of the distribution of HNRNPK binding across genes, its localization was mapped +/− 5 kb of the transcriptional start site of genes. HNRNPK binding was also compared to histone marks from the ENCODE consortium data set for human keratinocytes^40^ (Fig. 4c). Interestingly, HNRNPK binding correlated with marks of open chromatin and active transcription including RNA Pol II, H3K4me3, DNase I HSS, and H3K27ac while being depleted from closed/repressive chromatin marks such as H3K27me3 (Fig. 4c). Quantitation of HNRNPK binding +/− 5 kb from the TSS also showed that HNRNPK signals were broadly distributed throughout the region which was similar to RNA Pol II binding (Fig. 4d). In contrast, DNase I HSS sites accumulated at the TSS with diminished signal away from the TSS (Fig. 4d). Analysis of all HNRNPK bound peaks also showed HNRNPK correlated the most with RNA Pol II binding (Supplementary Fig. 4).

**Fig. 5:**
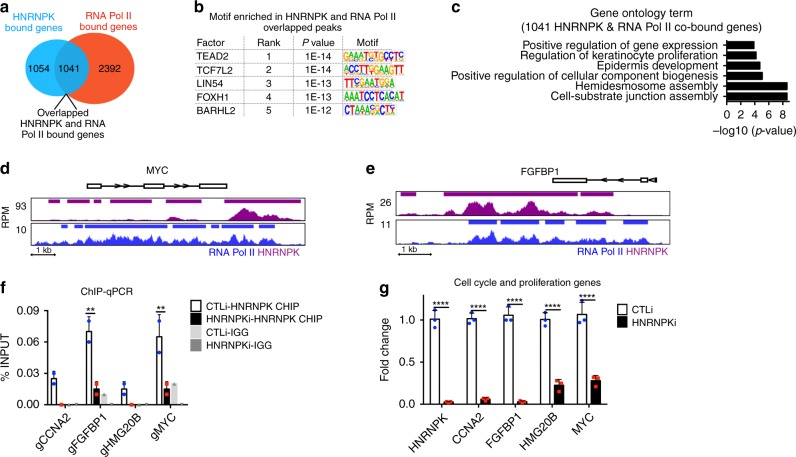
HNRNPK and RNA Pol II co-bind proliferation genes enriched in TEAD2 and TCF4 binding motifs. **a** Venn diagram showing overlap of genes bound by both HNRNPK and RNA Pol II. **b** De novo motif search for HNRNPK and RNA Pol II co-bound peaks. **c** Gene ontology analysis of the 1041 overlapped genes from **a**. **d**, **e** Gene tracks showing HNRNPK (purple) and RNA Pol II (blue) binding to MYC and FGFBP1 genomic regions. The *x*-axis shows genomic position and *y*-axis denotes signal strength (RPM, reads per million). Bars over peaks indicate significantly bound peaks with cutoff *q*-value of 0.05. **f** ChIP was performed on CTLi (white bar) and HNRNPKi (black bar) cells using a HNRNPK antibody. ChIP was also performed using IGG in CTLi (light gray bar) and HNRNPKi (dark gray bar) cells as a specificity control. HNRNPK binding to each gene was calculated as a percentage of input. *n* = 2. **g** RT-QPCR for mRNA expression of genes that are co-bound by HNRNPK and RNA Pol II in CTLi and HNRNPKi cells. QPCR results were normalized to *L32* levels. Mean values are shown with error bars = SD. *n* = 3 independent experiments performed in Fig. 5 unless otherwise indicated **p < 0.01, ****p < 0.0001 (2 way ANOVA followed by Tukey’s multiple comparison test for **f**. *T*-test for **g**)

Since HNRNPK binding was the most similar to RNA Pol II, we performed RNA Pol II ChIP-Seq on primary human keratinocytes in order to directly compare our HNRNPK ChIP-Seq data. RNA Pol II bound to 9485 peaks which mapped back to 3433 genes (Supplementary Data 3b). Interestingly, ~50% (1041/2095) of the genes HNRNPK bound to also had RNA Pol II binding (Fig. 5a). A search for transcription factor binding motifs on the HNRNPK and RNA Pol II co-bound peaks showed enrichment for TEAD2, TCF7L2, LIN54, FOXH1, and BARHL2 (Fig. 5b). The top 2 enriched motifs, TEAD2 and TCF7L2 (also known as TCF4), have been shown to be essential for epidermal stem cell self-renewal^13,41^ (Fig. 5b). The 1041 genes co-bound by HNRNPK and RNA Pol II were also enriched for GO terms such as epidermis development, hemidesmosome assembly, and regulation of keratinocyte proliferation (Fig. 5c). These regulators of keratinocyte proliferation included critical self-renewal and proliferation genes such as *MYC*, *FGFBP1*, and *CCNA2* (Fig. 5d, e, Supplementary Data 3). At each of these sites, HNRNPK bound in a similar manner as RNA Pol II (Fig. 5d, e). Knockdown of HNRNPK also decreased binding of HNRNPK to those genomic regions demonstrating the specificity of HNRNPK binding to the genomic regions of *MYC*, *FGFBP1*, *HMG20B*, and *CCNA2* (Fig. 5f). Loss of HNRNPK binding also resulted in decreased expression of those genes (Fig. 5f, g).

**Fig. 6:**
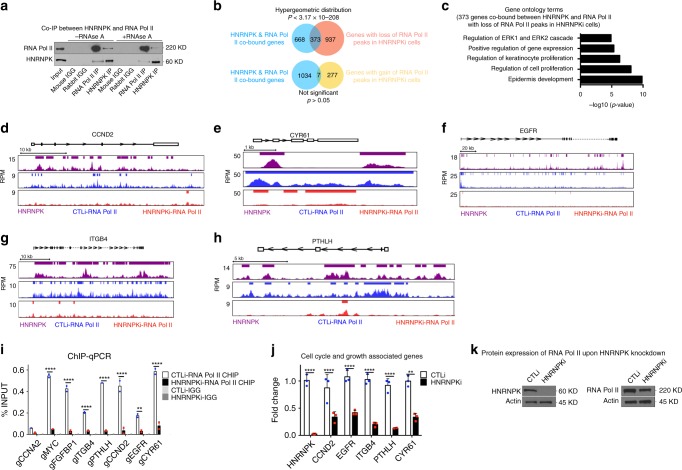
HNRNPK is necessary for RNA Pol II to bind proliferation genes to maintain the proliferative capacity of epidermal progenitor cells. **a** Immunoprecipitations (IPs) were performed using either an HNRNPK, RNA POL II antibody or IGG and Western blotted for HNRNPK or RNA Pol II protein expression. IPs were performed +/− RNase A. Five percent of the cell lysate was used as input. Representative blots are shown. *n* = 3. **b** Venn diagram showing the 1041 HNRNPK and RNA Pol II co-bound genes that lose RNA Pol II peaks upon HNRNPK depletion (top). The bottom Venn diagram depicts the 1041 HNRNPK and RNA Pol II co-bound genes that gain RNA Pol II peaks upon HNRNPK knockdown. **c** Gene ontology analysis of the 373 genes bound by both HNRNPK and RNA Pol II that lose RNA Pol II peaks upon HNRNPK knockdown. **d**–**h** Gene tracks showing HNRNPK (purple) and RNA Pol II binding to CCND2, CYR61, EGFR, ITGB4, and PTHLH genomic regions. RNA Pol II binding is shown in CTLi (blue) and HNRNPKi (red) cells. The x-axis shows genomic position and *y*-axis denotes signal strength (RPM, reads per million). Bars over peaks indicate significantly bound peaks with cutoff *q*-value of 0.05. **i** ChIP was performed on CTLi (white bar) and HNRNPKi (black bar) cells using a RNA Pol II antibody. ChIP was also performed using IGG as a specificity control in CTLi (light gray bar) and HNRNPKi (dark gray bar) cells. RNA Pol II binding to each gene was calculated as a percentage of input. *n* = 2. **j** RT-QPCR for mRNA expression of genes that are co-bound by HNRNPK and RNA Pol II, as well as lose RNA Pol II binding upon HNRNPK depletion. QPCR results were normalized to *L32* levels. *n* = 3. **k** Western blot analysis of RNA Pol II protein levels upon HNRNPK knockdown. Mean values are shown with error bars = SD. *n* = 3. **p < 0.01, ****p < 0.0001 (2 way ANOVA followed by Tukey’s multiple comparison test for 6**i**, 6**j**). Overlap significance in Venn diagrams was determined using hypergeometric distribution *p*-values (**b**)

Because half (1,041/2,095) of HNRNPK bound genes had RNA Pol II enrichment, it may suggest that HNRNPK is necessary for RNA Pol II to bind a subset of sites in the genome. In support of this, RNA Pol II can be found in the immunoprecipitate of HNRNPK and vice versa suggesting that these two proteins can associate with each other (Fig. 6a). This association was also dependent on the presence of RNA since RNAse A treatment diminished the interaction (Fig. 6a). To test if HNRNPK is necessary for RNA Pol II binding to HNRNPK bound sites, RNA Pol II ChIP-Seq was performed on HNRNPK knockdown cells. diffReps was used to determine the differential RNA Pol II peaks between control and knockdown cells^42^. These peaks were mapped back to their nearest genes which accounted for 1,310 genes with diminished/lost RNA Pol II binding upon HNRNPK knockdown while 284 genes gained/increased RNA Pol II binding (Fig. 6b, Supplementary Data 4). Incredibly, 36% (373/1041) of the HNRNPK and RNA Pol II co-bound genes lost RNA Pol II binding and resulted in diminished gene expression upon HNRNPK knockdown (Fig. 6b, k and Supplementary Fig. 5a). These genes were enriched for GO terms such as regulation of keratinocyte proliferation suggesting that HNRNPK is necessary for RNA Pol II association with proliferation genes to sustain the proliferative capacity of the epidermis (Fig. 6c). These genes include *CCND2*, *CYR61*, *EGFR*, *ITGB4*, *PTHLH*, *MYC*, and *FGFBP1* where loss of HNRNPK diminishes the ability of RNA Pol II to bind those regions (Fig. 6d–h, Supplementary Data 4). Validation of these results by ChIP-QPCR demonstrate that HNRNPK is necessary for RNA Pol II binding to these genes (Fig. 6i). Loss of RNA Pol II binding also led to the downregulation of each of these genes (Figs. 5g, 6j).

There were 668 genes co-bound by HNRNPK and RNA Pol II that did not significantly lose RNA Pol II binding upon HNRNPK knockdown. To analyze if HNRNPK was impacting RNA Pol II at these sites, the mean density profiles of RNA Pol II binding across the 668 genes were compared between control and HNRNPKi cells. Despite not being significant there was diminished RNA Pol II binding across these 668 genes upon HNRNPK knockdown (Supplementary Fig. 5b). This suggests that HNRNPK presence is required for RNA Pol II binding to HNRNPK bound genes.

It is also possible that the loss of RNA Pol II from the proliferation genes is due to the premature differentiation phenotype of HNRNPK knockdown cells rather than a direct requirement for HNRNPK presence in order for RNA Pol II to load onto the genes. To distinguish between these possibilities, HNRNPK and KLF4 double knockdown experiments were performed to prevent the premature differentiation phenotype of HNRNPK knockdown alone (Fig. 2h). In control cells, RNA Pol II bound to each of the proliferation genes whereas in HNRNPK knockdown cells the binding diminished (Supplementary Fig. 5c). In HNRNPK and KLF4 double knockdown cells, the RNA Pol II binding was similar to HNRNPK knockdown cells and significantly reduced compared to controls (Supplementary Fig. 5c). This suggests that loss of RNA Pol II binding to proliferation genes is not just due to the differentiation status of the cells but rather a requirement for HNRNPK binding.

**Fig. 7:**
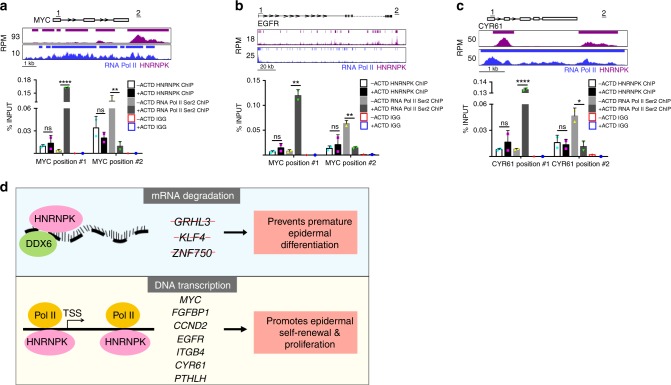
HNRNPK binding to proliferation genes is not dependent on active transcription and model of HNRNPK regulation of epidermal stem and progenitor cell self-renewal through mRNA transcription and degradation. **a**–**c** Keratinocytes were treated +/− actinomycin D (ACTD) to inhibit transcription. ChIP was performed using a HNRNPK or RNA Pol II CTD phospho Ser2 antibody in +/− actinomycin D (ACTD) treated cells. ChIP was also performed using IGG in +/− ACTD treated cells as a specificity control. Primers for each gene was designed at the 5’ end (genomic region #1) and past the 3’ end (genomic region #2) of each proliferation gene (*MYC*, *EGFR*, and *CYR61*). Binding to each gene was calculated as a percentage of input. Mean values are shown with error bars = SD, *p < 0.05, **p < 0.01, ****p < 0.0001, one-way ANOVA with Tukey’s multiple comparison test (**a**–**c**). *n* = 2. **d** Top panel: HNRNPK recruits DDX6 to degrade mRNAs that code for potent differentiation promoting transcription factors to prevent premature differentiation of epidermal cells. Bottom panel: HNRNPK recruits RNA polymerase II to self-renewal and proliferation genes to promote epidermal self-renewal

While HNRNPK is required for RNA Pol II loading onto proliferation genes, it is not clear if active transcription of the proliferation genes is necessary for HNRNPK binding to the genes. To test this, keratinocytes were treated +/− with Actinomycin D to block transcription. As a validation that the Actinomycin D blocked transcription, RNA Pol II CTD phospho Serine 2 (Ser2) ChIP was also performed. RNA Pol II CTD phospho Ser2 recognizes the elongating form of RNA polymerase. Primers were built at the TSS and past the 3’end of each gene. In all 3 proliferation genes tested, Actinomycin D treatment resulted in a dramatic decrease in RNA Pol II phospho Ser2 signal from the 3’ end of the gene and a buildup of signal at the TSS (Fig. 7a–c). This is in line with prior reports on how Actinomycin D inhibits transcription by causing the accumulation of CTD phosphorylated RNA Pol II at the TSS^43,44^. Importantly, blockade of transcription with Actinomycin D had no impact on HNRNPK binding to both the TSS and the 3’ end of MYC, EGFR, or CYR61 (Fig. 7a–c). This suggests that active RNA transcription is not required for HNRNPK binding to these proliferation genes and thus HNRNPK association with the genome is not co-transcriptional. Furthermore, there is only an overlap of 184 genes bound by HNRNPK on both the RNA and DNA level with enriched GO terms such as regulation of transcription (Supplementary Fig. 5d, e). These data support a model where HNRNPK’s genomic and mRNA targets are for the most part different.

Since HNRNPK is localized throughout the gene body and past the 3’end of genes, it may be possible that it is also regulating other aspects of transcription such as elongation or termination. Transcription elongation is controlled through cyclin T and CDK9 which form P-TEFb to phosphorylate Serine 2 on RNA Pol II CTD to promote elongation. In leukemia cell lines, HNRNPK and CDK9 can associate^45^. However, in keratinocytes no association between cyclin T or CDK9 could be detected with HNRNPK (Supplementary Fig. 6a, b). HNRNPK associates with the transcription termination factor, XRN2 to play a role in termination of *EGR1* in HCT116 cancer cell lines^46^. In keratinocytes, HNRNPK did not associate with XRN2 (Supplementary Fig. 6c). If HNRNPK regulated transcription elongation then loss of its expression may lead to accumulation of mRNA at the 5’ end of regulated genes. Similarly, if HNRNPK regulates transcription termination, then knockdown of HNRNPK may lead to readthrough transcription due to failure to properly terminate transcription as has been reported for loss of gene function for XRN2^47^ and HNRNPK^46^ in other systems. To determine potential HNRNPK impacts on either transcription elongation or termination, RNA-Seq data for the proliferation genes were analyzed. Notably, none of the proliferation genes had accumulation of transcripts past the 3’ end of the genes or accumulation at the 5’ end of the genes (Supplementary Fig. 7). In addition, there were no accumulation of RNA Pol II at the 5’ or 3’ end of HNRNPK targeted proliferation genes upon HNRNPK knockdown (Fig. 6d–h).

## Discussion

HNRNPK has been extensively studied for its role in tumorigenesis where it can act both as a tumor suppressor or oncogene depending on context^23,48^. Understanding the mechanisms for how HNRNPK either promotes or inhibits tumors have been more difficult to address due to the sheer number of cellular processes that it controls. The clear impact of HNRNPK on tumor growth and differentiation suggests that HNRNPK under normal circumstances may be a potent regulator of tissue homeostasis. Progress into this area has been hampered by embryonic-lethal phenotypes in Hnrnpk knockout mouse models^26^. Hnrnpk^+/−^ mice showed diminished survival with high penetrance of cancer phenotypes such as myeloproliferation, lymphomas, and hepatocellular carcinomas suggesting that Hnrnpk is necessary for inhibiting proliferation and promoting differentiation in those cell types^26^.

Interestingly, we find an opposite role for HNRNPK in epidermal cells where it is necessary to maintain cell proliferation while inhibiting premature differentiation. To explore how HNRNPK maintains stem and progenitor cell status, we used RIP-Seq to define the transcripts that HNRNPK directly binds. Notably, HNRNPK bound to 3 of the most important genes known that code for differentiation inducing transcription factors such as GRHL3, KLF4, and ZNF750. We and others have previously published that these factors are absolutely required for promoting epidermal differentiation^4,5,7^. Knockdown of HNRNPK led to increased transcript stability and thus increased the mRNA levels of these genes. This suggests that HNRNPK normally functions in stem and progenitor cells to bind and degrade these transcripts to prevent premature differentiation. Supporting this, double knockdown of HNRNPK with KLF4 or GRHL3 can partially reverse the premature differentiation phenotype of just HNRNPKi alone suggesting that these transcription factors are the key factors downstream of HNRNPK that promotes differentiation.

The next question we addressed was how HNRNPK promotes the degradation of differentiation transcripts. Our prior work suggested that the RNA helicase DDX6 which is part of the mRNA degradation complex with enhancer of mRNA-decapping protein 3 (EDC3) binds and degrades mRNAs such as *KLF4* to prevent premature differentiation^14–16^. However it was unclear what was recruiting DDX6/EDC3 to these transcripts since these factors have no inherent RNA binding specificity. Here, we demonstrated that in the absence of HNRNPK, DDX6 could not bind to *KLF4*, *GRHL3*, or *ZNF750* mRNAs, whereas HNRNPK can still bind in the absence of DDX6. This suggests that HNRNPK is necessary for DDX6 to bind target mRNAs.

Besides its ability to regulate RNA, HNRNPK has also been described to bind DNA. It has been reported as both an activator and repressor of gene expression on the chromatin level. As a repressor, HNRNPK is necessary to recruit the PRC1 complex to Xist targeted regions to mediate chromosomal silencing^49^. As an activator, HNRNPK can bind promoter regions to facilitate transcription of genes such as c-myc^50^. In leukemic cell lines, HNRNPK is necessary to bring together lineage determining transcription factors with the transcriptional machinery to mediate 5-azacitidine sensitive chromatin structure^45^. HNRNPK has also been described to promote transcriptional termination through the XRN2 pathway^46^. Since HNRNPK binding to genomic DNA may potentially regulate genes involved in epidermal self-renewal, we focused on determining its genomic binding sites. HNRNPK binding tended to localize throughout the gene similar to RNA Pol II binding.

Surprisingly, half of the HNRNPK bound genes also had RNA Pol II binding which were specifically enriched in genes regulating keratinocyte proliferation and hemidesmosome formation. These co-bound genes were also enriched for TEAD2 and TCF7L2 (TCF4) transcription factor binding motifs. TEAD2 is part of the YAP1 signaling pathway absolutely essential for epidermal growth and TCF4 is necessary for epidermal stem cell self-renewal^13,41^. This suggests that HNRNPK/RNA Pol II may be acting downstream of these factors to promote the transcription of genes important for epidermal growth. In support of this, HNRNPK could associate with RNA Pol II through RNA in co-immunoprecipitation experiments. Knockdown of HNRNPK led to the depletion of RNA Pol II from 36% of the HNRNPK and RNA Pol II co-bound genes many of which coded for proliferation genes. The loss of RNA Pol II binding on proliferation genes upon HNRNPK knockdown is not just a consequence of the cells prematurely differentiating as blocking the differentiation phenotype by HNRNPK and KLF4 double knockdown did not restore RNA Pol II binding. This suggests that RNA Pol II loading onto HNRNPK bound genes is dependent on the presence of HNRNPK. These genes that require HNRNPK for proper RNA Pol II localization include growth signaling molecules (*FGFBP1*, *EGFR*, *PTHLH*, *CYR61*), proliferation cyclins (*CCND2*, *CCND1*), transcription factors promoting proliferation (*MYC*, *FOSL1*), and hemidesmosome assembly (*ITGB4*, *LAMB3*, *LAMC2*, *LAMA3*). In contrast, HNRNPK presence on proliferation genes was not dependent on active transcription of the proliferation genes since inhibition of transcription with Actinomycin D had no impacts on HNRNPK genomic localization.

Given the multitude of functions for HNRNPK, it is also possible that HNRNPK regulates other aspects of transcription such as through elongation or termination to control the expression of proliferation genes. HNRNPK has been reported to associate with CDK9 and XRN2 in cancer cells to potentially regulate transcription elongation and termination, respectively^45,46^. However, we did not find clear evidence for this mode of regulation in keratinocytes. HNRNPK did not associate with either cyclin T or CDK9. HNRNPK depletion also resulted in diminished RNA Pol II binding throughout the entire gene rather than RNA Pol II stalling/buildup at the 5’ end which would be indicative of alterations in transcription elongation. Similarly, HNRNPK did not associate with XRN2 and there were no detectable alterations in transcription termination.

HNRNPK may also be loading RNA Pol II onto genes through binding enhancers. Twenty-one percent of HNRNPK binding sites are found in intergenic regions and its sites are enriched for YAP1 signaling pathway components which are known to bind enhancers^51^. This may be an area of future investigation.

HNRNPK’s mechanism of function has been difficult to decipher due to its role in numerous cellular processes. To our knowledge, we are the first to comprehensively determine its targets in a high-throughput manner on the RNA and DNA levels, as well as characterize its impacts on adult tissue self-renewal. Furthermore, we have described 2 separate mechanisms by which HNRNPK mediates epidermal self-renewal and prevention of premature differentiation through both mRNA degradation and transcriptional activation (Fig. 7d). It is also interesting to note that our results suggest a previously unknown mode of regulation where HNRNPK is necessary for RNA Pol II to drive the expression of potent regulators of growth and self-renewal.

In summary, our findings describe a novel mechanism for HNRNPK regulated tissue self-renewal through both transcriptional and post-transcriptional mechanisms.

## Methods

### Cell culture

Primary human epidermal keratinocytes were derived from neonatal foreskin and cultured in EpiLife medium (ThermoFisher: MEPI500CA) mixed with human keratinocyte growth supplement (HKGS, ThermoFisher: S0015) and pen/strep^11,14,33^. Phoenix cells (ATCC CRL-3214) were cultured in DMEM with 10% fetal calf serum.

### Knockdown of genes

To knockdown HNRNPK stably, retroviruses expressing HNRNPK shRNAs were used. The retroviral constructs (3 µg) were transfected using Lipofectamine 2000 (Life Technologies: 11668027) into amphotropic phoenix cells. Viral supernatants were collected 48 h post-transfection and used to infect primary human keratinocytes^33^. Cells were incubated in the viral supernatants and centrifuged at 1000 rpm for 1 h with hexadimethrine bromide (Sigma-Aldrich: H9268). Cells were transduced on two consecutive days. Cells were selected in puromycin 24 h after the last transduction to select for cells stably expressing the shRNAs (the retroviral vector encodes a puromycin resistance gene). shRNA retroviral constructs were generated by cloning oligos into the pSuper retroviral vector^14,52^. The shRNA sequence targeting HNRNPK is GGTTTCAGTGCTGATGAAA. The scrambled control shRNA sequence is GATACTGACTACCAAGGAT and cloned into the pSuper retroviral vector. siRNAs targeting HNRNPK, GRHL3, KLF4, CDKN1A and control siRNAs were purchased from Dharmacon. The following siRNA sequences were used to target the following genes: HNRNPK: GTCGGGAGCTTCGATCAAA; GRHL3: CATCAAGTCAGGCGAGTCA, CCACAGGAGTCGATGCTCT, CCAACAAAGTCAAGAGTGT, and TTGAGGAGGTGGCCTATAA; KLF4: TGACCAGGCACTACCGTAA; CDKN1A: GATGGAACTTCGACTTTGT, GCGATGGAACTTCGACTTT, CGATGGAACTTCGACTTTG, and CGACTGTGATGCGCTAATG.

### Organotypic cultures and in vivo competition assays

For the organotypic skin cultures, one million control or HNRNPK knockdown cells were seeded onto devitalized human dermis to regenerate human epidermis^11,31,33^. Human dermis was purchased from the New York Firefighters Skin Bank. Dermis seeded cells were raised to the air liquid interface to promote differentiation and stratification. Tissue was harvested 3 days after initial seeding. For the in-vivo epidermal progenitor cell competition assay, primary human keratinocytes were first transduced with a retrovirus encoding GFP or dsRED. The GFP or dsRED cells were than infected with retroviruses encoding control (CTLi) or HNRNPK (HNRNPKi) shRNAs^14,33,34^. GFP-CTLi cells were mixed at a 1:1 ratio with dsRED-HNRNPKi cells. dsRED-CTLi cells were also mixed with GFP-HNRNPKi cells at equal ratios to ensure results were not due to the influence of fluorescence proteins. A total of one million cells were seeded on the devitalized human dermis to regenerate human epidermis. The regenerated human epidermis was then grafted on immune compromised mice (NOD SCID Gamma/NSG) purchased from Jackson Labs. Three and eighteen days post-grafting, the human skin grafts were harvested from the mice. The human skin grafts were fixed in 4% paraformaldehyde for 1 h and embedded into OCT compound for sectioning. The contribution of GFP and dsRED expressing cells to the epidermis was quantified as a percentage of GFP or dsRED positive cells divided by the combined number of GFP and dsRED cells in the epidermis. Analysis was performed using ImageJ. Ten independent sections each from three skin grafts per group per timepoint were imaged and quantified. All animal work was conducted in accordance with UCSD’s IACUC guidelines.

### Measurement of mRNA stability and half-life

Control and HNRNPK knockdown cells were treated with actinomycin D (10 µg/mL) for 0, 0.5, 1, 2, 4, and 6 h to determine the half-lives of *CDKN1A*, *GRHL3*, *KLF4*, and *ZNF750* mRNAs. RNA was isolated from the samples and RT-QPCR was used to determine the levels of respective mRNAs. Half-lives was calculated using the formula T_1/2_ = 0.3t/log(D1/D2)^14,33,53^.

### Apoptosis assay

Control and HNRNPK knockdown cells were stained with Annexin V conjugated to Alexa Fluor 488 (Life Technologies: A13201) and analyzed using the Guava flow cytometer (Millipore) according to manufacturers instructions^14^.

### RNA isolation and RT-QPCR

Total RNA from cells or tissue was extracted using the GeneJET RNA purification kit (Thermo Scientific: K0732) and quantified using a Nanodrop. One µg of total RNA was reversed transcribed using the Maxima cDNA synthesis kit (Thermo Fisher: K1642). Quantitative PCR was performed using the Roche 480 Light Cycler. *L32* was used as internal control for normalization. Primer sequences for *GAPDH*, *LCE3D*, *LOR*, *KLF4*, *ZNF750*, and *GRHL3* were previously published^10,14,32–34^ and are as follows: GAPDH forward: CTGAGAACGGGAAGCTTGT, GAPDH reverse: GGGTGCTAAGCAGTTGGT; LCE3D forward: GCTGCTTCCTGAACCAC, LCE3D reverse: GGGAACTCATGCATCAAG; LOR forward: CCGGTGGGAGCGTCAAGT, LOR reverse: AGGAGCCGCCGCTAGAGAC; KLF4 forward: GCCTCCTCTTCGTCGTC, KLF4 reverse: GGCTCACGTCGTTGATGT, ZNF750 forward: AGCTCGCCTGAGTGTGAC, ZNF750 reverse: TGCAGACTCTGGCCTGTA; GRHL3 forward: GCCAGTTCTACCCCGTCA, GRHL3 reverse: GTCAATGACCCGCTGCTT. Sequences for *L32*, *FLG*, *TGM1*, *K1*, *K10*, *CDKN1A*, *HMG20B*, *CCNA2*, *CDK4*, *MKI67*, *MYC*, *FGFBP1*, *HNRNPK*, *PTHLH*, *CCND2*, *ITGB4*, *CYR61*, and *EGFR* are as follows: L32 forward: AGGCATTGACAACAGGGTTC, L32 reverse: GTTGCACATCAGCAGCACTT; FLG forward: GGCAAATCCTGAAGAATCCA, FLG reverse: TGCTTTCTGTGCTTGTGTCC; TGM1 forward: TCAGACGCTGGGGAGTTC, TGM1 reverse: GGTCCGCTCACCAATCTG; K1 forward: TACCTCCACTAGAACCCAT, K1 reverse: GCTGCAAGTTGTCAAGTT; K10 forward: CGCCTGGCTTCCTACTTGG, K10 reverse: CTGGCGCAGAGCTACCTCA; CDKN1A forward: CCTTCCCATCGCTGTCAC, CDKN1A reverse:TCACCCTGCCCAACCTTA; HMG20B forward: ACGCGCTACACTGGCTCT, HMG20B reverse: CCACCCATCTGGGGTACA; CCNA2 forward: AGACGAGACGGGTTGCAC, CCNA2 reverse: AAAGCCAGGGCATCTTCA; CDK4 forward: GCTGCCTCCAGAGGATGA, CDK4 reverse: GCTGCAGAGCTCGAAAGG; MKI67 forward: TGGCCAAGAACGCCTAAG, MKI67 reverse: GGCCATTGCTTTGTGCTT; MYC forward: CGGACACCGAGGAGAATG, MYC reverse: GCTTGGACGGACAGGATG; FGFBP1 forward: TCTGGGCAACACCCAGAT, FGFBP1 reverse: GGCATGAGGTTGGATTGC; HNRNPK forward: AAGAATGCTGGGGCAGTG, HNRNPK reverse: CAGGCCCTCTTCCAAGGT; PTHLH forward: GATGCAGCGGAGACTGGT, PTHLH reverse: GGAAGAATCGTCGCCGTA; CCND2 forward: GCTCCAGCAGGATGAGGA, CCND2 reverse: CCGACTTGGATCCGTCAC; ITGB4 forward: AGGCCCAAGCTGTGACTG, ITGB4 reverse: AGCGTAGGTCCTCGCAGA; CYR61 forward: GGTTTCCAGGGCACACCT, CYR61 reverse: AGTGTCCATCCGCACCAG; EGFR forward: GGGCCGACAGCTATGAGA, EGFR reverse: GGCAGGATGTGGAGATCG.

### Western blotting

Twenty microgram of the cell lysates were used for immunoblotting and resolved on 10% SDS-PAGE and transferred to PVDF membranes. Primary antibodies used include beta-actin (Santa Cruz: sc-47778) at 1:5000, HNRNPK (Bethyl Laboratories: A300–674A) at 1:3000, DDX6 (Novus Biologicals: NB200–192) at 1:2000, RNA Pol (Active motif: 39097) at 1:4000, Cyclin T1 (Bethyl Laboratories: A303–499A) at 1:1000, XRN2 (Cell Signaling: 13760) at 1:1000 and CDK9 (Bethyl Laboratories: A303–493A) at 1:1000. Secondary antibodies including donkey anti-rabbit IgG HRP (Sigma: NA934V) and goat anti-mouse IgG-HRP (Santa Cruz: sc-2005) were used at 1:2000. All original blots can be found in the accompanying Source Data.

### Histology and immunofluorescence

Cultured cells or tissue were fixed in 4% paraformaldehyde for 11 min followed by blocking in PBS with 2.5% normal goat serum, 0.3% triton X-100, and 2% bovine serum albumin for 30 min Primary antibodies used were Keratin 1 (Biolegend: PRB-149P) at 1:1000, Filaggrin (Abcam: ab3137) at 1:200, MKi67 (Abcam: ab16667) at 1:300, Keratin 10 (Abcam: ab9025) at 1:500, HNRNPK (Bethyl Laboratories: A300–674A) at 1:1000 for 1 h. The secondary antibodies used were Alexa 555 conjugated goat anti-mouse IgG (ThermoFisher: A11029) or Alexa 488 conjugated donkey anti-rabbit IgG (ThermoFisher: A21206) both at 1:500. Nuclear dye, Hoechst 33342 was used at 1:1000 (ThermoFisher: H3570).

### RNA-seq and bioinformatics analysis

Control or cells knocked down for HNRNPK were harvested 6 days after the last infection. Two technical duplicates were obtained for both CTLi and HNRNPKi and total RNA was isolated using the GeneJET RNA purification kit (Thermo Scientific: K0732) and quantified by Nanodrop. RNA-seq was performed using the Illumina Hi Seq 4000 machine at the Institute of Genomic Medicine core facility at UCSD. RNA-seq libraries were prepared with TruSeq RNA Library Prep Kit (Illumina: RS-122–2001) then multiplexed and ~40 million reads per sample were obtained. Reads were aligned to the GENCODE v19 transcriptome hg19 using TopHat2 with default settings^54^. Differential expression among samples was calculated using ANOVA from the Partek Genomic Suite (Partek Incorporated). Analysis of the read count distribution indicated that a threshold of ten reads per gene generally separated expressed from unexpressed genes, so all genes with fewer than ten reads were excluded from ANOVA analysis. Gene lists for significantly upregulated or downregulated genes were created using *p* < 0.05 and 2-fold change. Enriched GO terms for RNA-seq differentially expressed gene sets were identified using Enrichr^55,56^. Heatmaps for the RNA-seq data were generated using Partek’s Genomic Suite (http://www.partek.com/partek-genomics-suite/).

### RNA immunoprecipitation/high-throughput sequencing (RIP-Seq)

Three million control, HNRNPK, or DDX6 knockdown cells and 3 µg of each antibody were used for each pulldown experiment. The following antibodies were used for RNA-IP: HNRNPK (Bethyl Laboratories: A300–674A), DDX6 (Novus Biologicals: NB200–192) and rabbit IgG control (Millipore: 12–370). RNA-IP was performed using the Magna RIP RNA IP kit (17–700) from Millipore according to manufacturer’s protocol. The same volume of immunoprecipitated RNA was converted into cDNA using the Maxima cDNA synthesis kit (Thermo Fisher: K1642). RT-QPCR was used to determine the amount of RNA bound and presented as a percentage of input. For RIP-Seq, 3 million proliferating primary human keratinocytes were IP’d using 3ug of the HNRNPK antibody or 3 µg of IgG using the Magna RIP RNA IP kit. The eluted immunoprecipitated RNA from the HNRNPK antibody pulldown or with IgG was subject to sequencing (HiSeq400) with methods and analysis as described in the RNA-Seq and Bioinformatics Analysis section with the following modifications. RNA associated with HNRNPK was determined by comparing the HNRNPK pulldown samples to the IgG pulldown samples. Differential expression among samples was calculated using ANOVA from the Partek Genomic Suite (Partek Incorporated). Transcripts significantly enriched in the HNRNPK pulldowns were defined as ≥4 fold change over IgG and *p* < 0.05 (ANOVA). Heatmaps for the RIP-seq data were generated using Partek’s Genomic Suite (http://www.partek.com/partek-genomics-suite/).

### ChIP-qPCR and ChIP-Seq

Ten million cells and 5 µg of antibody were used for each antibody pulldown experiment for ChIP^32,34^. ChIP was performed using the following antibodies: HNRNPK (Bethyl Laboratories: A300-674A), RNA Pol II (Active motif: 39097), RNA Pol II CTD Phospho S2 (Active Motif: 91115), Rabbit IgG (Millipore: 12–370) and mouse IgG (Abcam: ab18413). Cells for the RNA Pol II ChIP-QPCR or ChIP-Seq were fixed at a final concentration of 1% formaldehyde (ThermoFisher 28908). Cells for the HNRNPK ChIP-QPCR or ChIP-Seq were fixed in both formaldehyde (1% final concentration) and disuccinimidyl glutarate (DSG, Thermo Fisher 20593, 2 mM final concentration). QPCR results are represented as a percentage of input DNA. QPCR primers for ChIP are as follows: CCNA2 FOR: AGTTGCCCAACATCACTGCT, CCNA2 REV: CGGCGGCTACGACTATTCT; FGFBP1 FOR: TCCCAGACACCTGACCTCTC, FGFBP1 REV: TGGAGCTGGATTTTGGAAAG; HMG20B FOR: CCCTGAGTCACCCCCTACC, HMG20B REV: GGGCCATGTAGAAGTCCAGA; MYC FOR: CAAAAATGAGGGGCTGTGTT, MYC REV: GGCAAGGATTTGCTTTTCAG; PTHLH FOR: ACCTGCAACAGAAGGGAATG, PTHLH REV: ACTTGGGAGATGCCCTTGAT; ITGB4 FOR: CCTTCTGTGCCTGGTCTCTC, ITGB4 REV: CCCACACTGTGACTGCCATA; CCND2 FOR: CCGAAAACCCCCTATTTAGC, CCND2 REV: CCCTCTCCCTCCTGCTTTC; EGFR FOR: AGGGAAGCTGAGGAAGGAAC, EGFR REV: CCGGCTTCAGTTTGAGACCT; CYR61 FOR: ACCAGCTTGTTGGCGTCTT, CYR61 REV: GGTCAAGTGGAGAAGGGTGA; MYC position 1 FOR: GGAGATCCGGAGCGAATAG, MYC position 1 REV: GCTGCTATGGGCAAAGTTTC; MYC position 2 FOR: GTCCCAAGCACTCCTAAGCA, MYC position 2 REV: CAGTGAATCTTGGGCATGTG; CYR61 position 1 FOR: ACCAGCTTGTTGGCGTCTT, CYR61 position 1 REV: GGTCAAGTGGAGAAGGGTGA; CYR61 position 2 FOR: AAGGTGTGAGGCTTTTGTGG, CYR61 position 2 REV: TTGTTGGACTCCAGTGTTGG; EGFR position 1 FOR: AGGGAAGCTGAGGAAGGAAC, EGFR position 1 REV: CCGGCTTCAGTTTGAGACCT; EGFR position 2 FOR: GGGAAAGGGTGTAGCCCATA, EGFR position 2 REV: TTCCTGTTGGGTTTTCAGGT.

For ChIP-Seq, the ChIP DNA library was prepared using the TruSeq DNA sample prep kit (Illumina). Sequencing was done on HiSeq 4000 System (Illumina) using single 1 × 75 reads at the Institute for Genomic Medicine Core, UCSD. HNRNPK ChIP-seq was performed in triplicates. The RNA Pol II ChIP-Seq was performed in CTLi and HNRNPKi cells in duplicates. The ChIP-seq reads were processed by the Kundaje ChIP-seq pipeline (https://github.com/kundajelab/chipseq_pipeline) on our local workstation. The reads were first trimmed based on quality score before alignment to reference hg19; Upon alignment and deduplication, the peak-calling was then carried out by MACS2^38,39^ with a cutoff q-value of 0.05. The heatmaps for the ChIP-Seq data were generated using seqMINER^57^. Gene tracks were visualized using UCSC genome browser along with annotation tracks. Differential peaks between samples were obtained by diffReps 1.55.4^42^ using negative binomial test with a scanning window size of 1000 bp, step size of 100 bp, and a cutoff *p*-value of 0.0001.

### Co-immunoprecipitation experiments

Cells were lysed in a non-denaturing IP buffer (25 mM Tris-HCl pH 7.4, 150 mM NaCl, 1 mM EDTA, 1% NP-40 and 5% glycerol). Three microgram of HNRNPK antibody (Bethyl Laboratories: A300–674A), DDX6 (Novus Biologicals: NB200–192), RNA Pol II (Active motif: 39097), CDK9 (Bethyl Laboratories: A303–493A) or rabbit IgG were complexed with 50 µl of Protein G Dynabeads (Life Technologies: 10004D) at room temperature for 30 min The antibody conjugated Dynabeads were then incubated with cell lysates on a rotator at 4 °C overnight. The next day, the supernatant was removed and the immunoprecipitated complex was washed with 500 µl of IP wash buffer for a total of 6 times at room temperature. Then 30 µl of RIPA buffer (25 mM Tris-HCl (pH 7.6), 150 mM NaCl, 1% NP-40, 1% sodium deoxycholate, 0.1% SDS) and 15 µl NuPAGE® LDS Sample Buffer (Life Technologies: NP0008) were used to elute at 70 °C. Immunoprecipitates were loaded onto SDS-PAGE gels for Western blot. For samples with RNase treatment, after the last wash of IP wash buffer, the immunoprecipitated complexes were then washed with PBS once and then equally dividing into two tubes in 1 mL of PBS. One tube was subjected to 100 µg/mL RNase A treatment for 1.5 h on ice. The immunoprecipitated complexes with or without RNase A treatment were washed three times with IP wash buffer before elution and blotted using Western blot^14^.

### Inhibition of transcription with actinomycin D

Proliferating primary human keratinocytes were treated +/− with Actinomycin D at 1ug/ml final concentration for 3 h before harvesting. Cells were harvested for ChIP as described in the “ChIP-qPCR and ChIP-Seq” section.

### Statistical analysis

Graph data are presented as mean ± SD. Statistical analyses were performed using GraphPad Prism. Student’s *t* tests and One-way ANOVA were used to compare between two or more groups, and significant changes were defined as *p* < 0.05. The number of biological experiments performed is indicated by N in the figure legends.

### Scatter plot of ChIP-Seq data comparisons

Replicate ChIP-Seq bam files of HNRNPK and RNA Pol II were merged using Samtools. Bam files of H3K27ac, H3K4me3, H3K36me3, DNAse I, and H3K27me3 were downloaded from the ENCODE database. Total count for each sample was obtained by Samtools. The read counts over the HNRNPK peak regions were then obtained using bedtools. RPKM for each peak region was computed using the following formula:

$${\rm{RPKM}} = \frac{{{\rm{read}}\;{\rm{count}} \times 10^9}}{{{\rm{total}}\;{\rm{read}}\;{\rm{count}} \times {\rm{peak}}\;{\rm{length}}}}$$

A RPKM cutoff (0.0001 for counts over HNRNPK peaks) was then applied to remove the low count peak regions followed by log2 transformation. R package ggplot2 was used to plot the scatter plot.

### Reporting summary

Further information on research design is available in the Nature Research Reporting Summary linked to this article.

## Supplementary information

Supplementary Information

Reporting Summary

Description of Additional Supplementary Files

Supplementary Data 1

Supplementary Data 2

Supplementary Data 3

Supplementary Data 4

Source Data

## Data Availability

The GEO accession number for RNA-Seq, RIP-Seq, and ChIP-Seq data reported in this paper is GSE122327.
