# Supplementary material for: HNRNPK maintains epidermal progenitor function through transcription of proliferation genes and degrading differentiation promoting mRNAs
Source: Nat Commun. 2019 Sep 13;10:4198. doi: 10.1038/s41467-019-12238-x (PMC6744489; doi:10.1038/s41467-019-12238-x)
Supplement: Supplementary file 1 — Supplementary Information [file 41467_2019_12238_MOESM1_ESM.pdf]

Supplementary information

HNRNPK maintains epidermal progenitor function through transcription of proliferation genes and degrading differentiation promoting mRNAs

Li et al.

Supplmentary Figures

Supplementary Figure 1

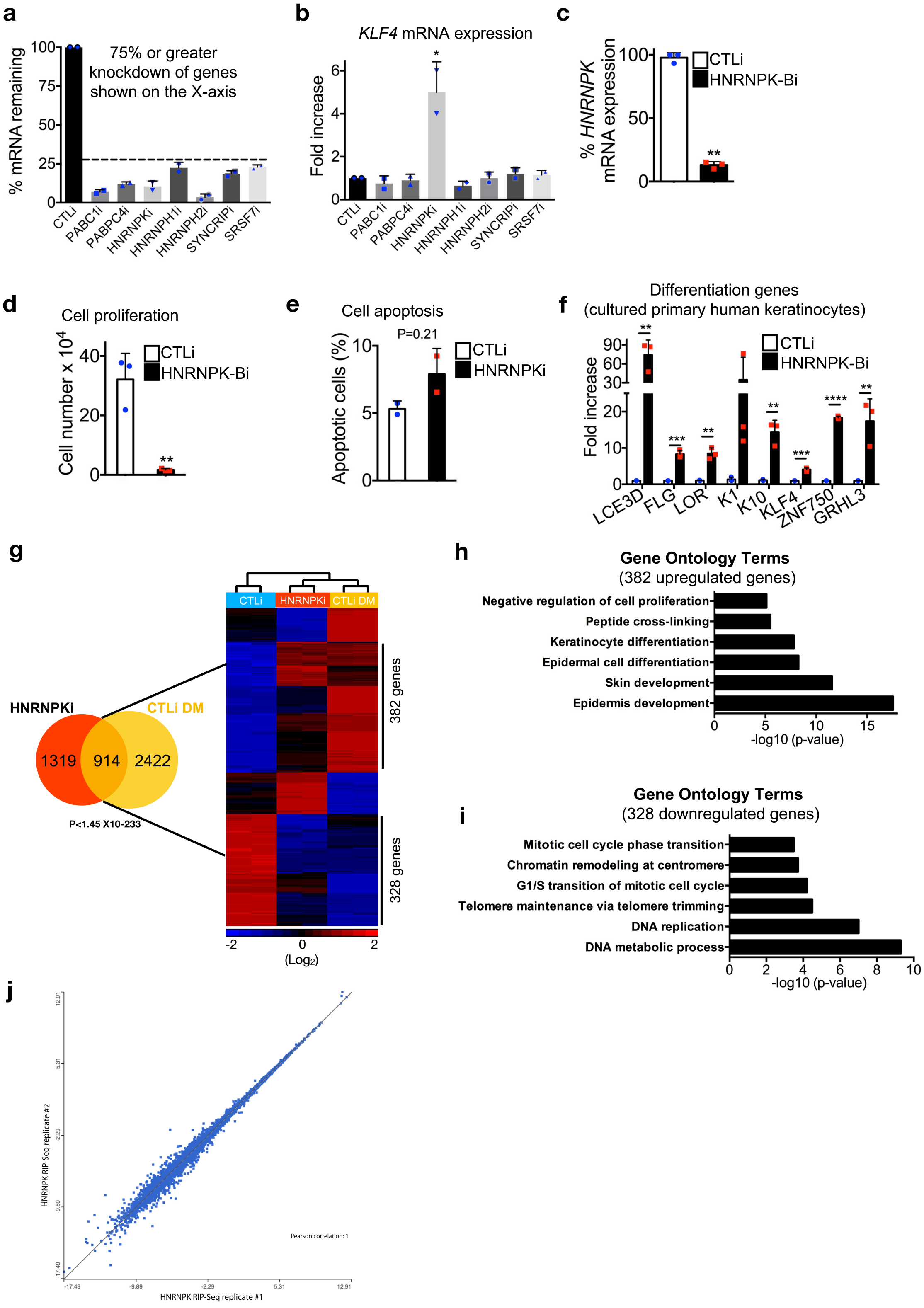

Supplementary Figure 1. HNRNPK Promotes Epidermal Self-renewal and Prevents Premature Differentiation. Supports Figures 1 and 2.
(a) A panel of RNA-binding proteins that we previously found to associate with DDX6 were knocked down in epidermal progenitor cells using siRNAs. Cells were cultured for 4 days and knockdown levels were assessed using RT-QPCR. Knockdown for each gene is shown on the X-axis. QPCR results were normalized to *L32* levels. N=2 **(b)** *KLF4* expression was measured by RT-QPCR in the knockdown cells. QPCR results were normalized to *L32* levels. N=2 **(c)** Use of another siRNA targeting a different sequence in HNRNPK (HNRNPK-Bi) to validate results. Knockdown levels of HNRNPK expression were determined by QPCR. **(d)** Cell proliferation counts were performed by seeding the same number of cells between CTLi and HNRNPK-Bi cells and counted 4 days after plating. **(e)** Percentage of apoptotic cells was determined by Annexin V staining in CTLi and HNRNPKi cells and quantitated using flow cytometry. N=2 **(f)** Differentiation marker expression in CTLi and HNRNPK-Bi cells were analyzed by RT-QPCR one week after plating. **(g)** Overlap (left panel) of the differentiation gene signature (CTL DM: 3,336 genes) with the genes that change upon knockdown of HNRNPK in cells cultured in growth medium (HNRNPKi: 2,233 genes). The differentiation gene signature (CTL DM) is the differentially expressed genes when epidermal cells are induced to differentiate in high calcium for 3 days. Heat map (right panel) of the 914 genes that overlap. Differentiated control cells (CTL DM) were compared to control (CTLi) and HNRNPK knockdown (HNRNPKi) cells cultured in growth conditions. Heat map is shown in red (induced genes) and blue (repressed genes) on a log 2-based scale. RNA-seq experiments were performed in biological duplicates. **(h)** Gene ontology terms of the 382 upregulated genes in both HNRNPKi and CTL DM samples. **(i)** Gene ontology terms of the 328 repressed genes in both HNRNPKi and CTL DM samples. **(j)** Pearson correlation of the replicate HNRNPK RIP-Seq data. N=3 independent experiments performed for all experiments unless otherwise indicated. All error bars=SD. *$\boldsymbol{p}$ <0.05, **$\boldsymbol{p}$ <0.01, ***$\boldsymbol{p}$ <0.001, ****$\boldsymbol{p}$ <0.0001 (T-test). Overlap significance in Venn diagrams was determined using hypergeometric distribution p-values.

Supplementary Figure 2

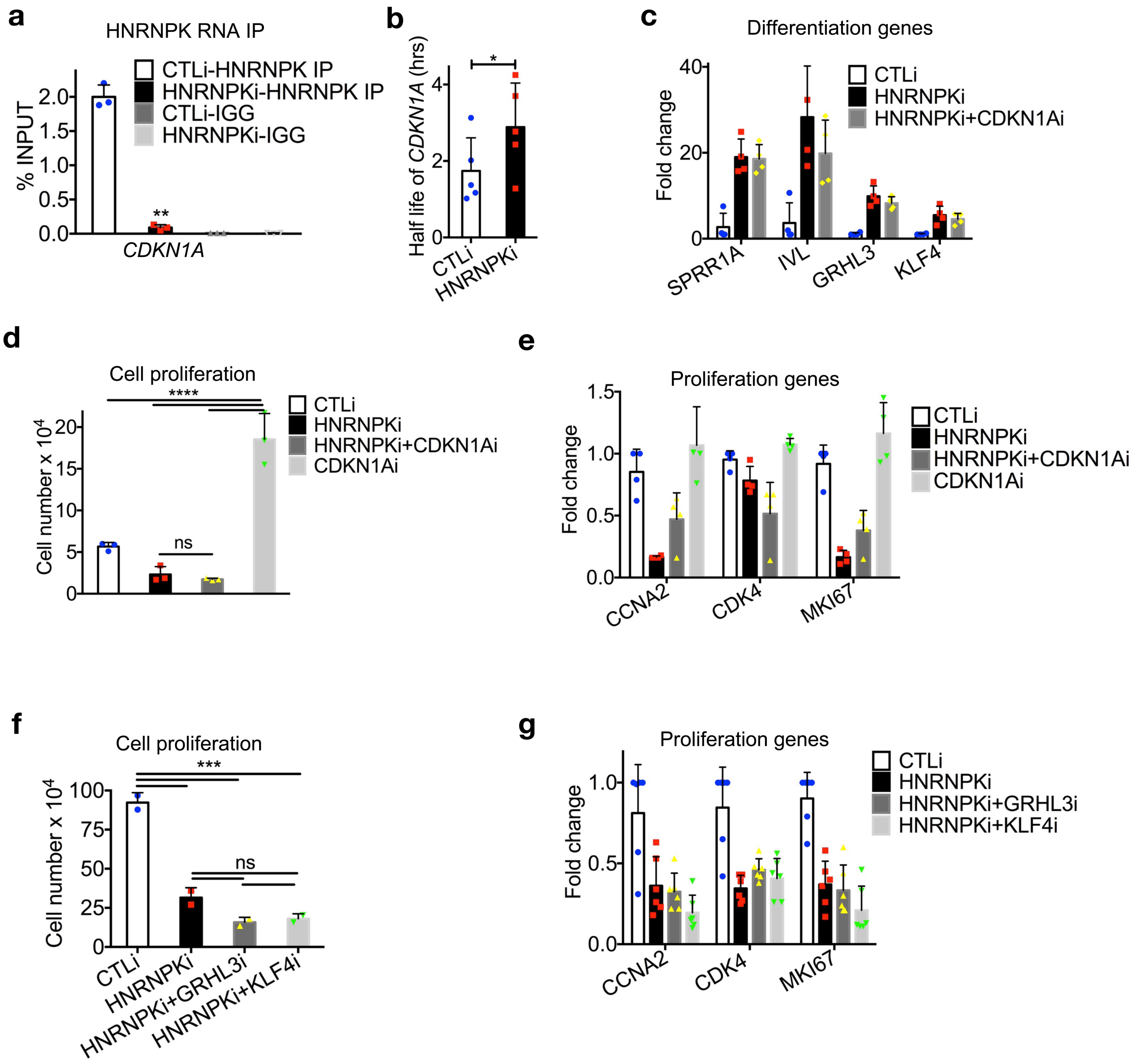

Supplementary Figure 2. The Impacts of HNRNPK Binding to *CDKN1A*, *GRHL3,* and *KLF4* Transcripts on Epidermal Growth and Differentiation. Supports Figure 2. **(a)** RNA IP was performed in CTLi and HNRNPKi cells using an HNRNPK antibody. RT-QPCR was used to determine the levels of binding between HNRNPK and *CDKN1A* mRNA in CTLi and HNRNPKi cells. IGG IPs in CTLi and HNRNPKi cells were used as specificity controls. Binding was calculated as a percent of input. **(b)** Half life of *CDKN1A* was measured by treating CTLi and HNRNPKi cells with actinomycin D. RT-QPCR was used to measure the levels of *CDKN1A*. N=5 **(c)** Double knockdown of HNRNPK with CDKN1A was performed and differentiation markers were evaluated by RT-QPCR. Cells were harvested 4 days after knockdown. N=4 **(d)** CTLi and knockdown cells were plated at the same number and counted 4 days later for all groups. **(e)** Proliferation related genes were assessed in CTLi, HNRNPKi, CDKN1Ai and double knockdown cells using QPCR. N=4 **(f)** CTLi, HNRNPKi, HNRNPKi+GRHL3i, and HNRNPKi+KLF4i cells were plated at the same number and counted 4 days later. N=2 **(g)** Proliferation related genes were assessed in CTLi, HNRNPKi, HNRNPKi+GRHL3i, and HNRNPKi+KLF4i cells using QPCR (N=6). N=3 independent experiments performed for all experiments unless

otherwise indicated. All error bars=SD. **p <0.01, ***p <0.001, ****p <0.0001 (One way ANOVA for 2d, 2f. T-test for 2a, 2b).

Supplementary Figure 3

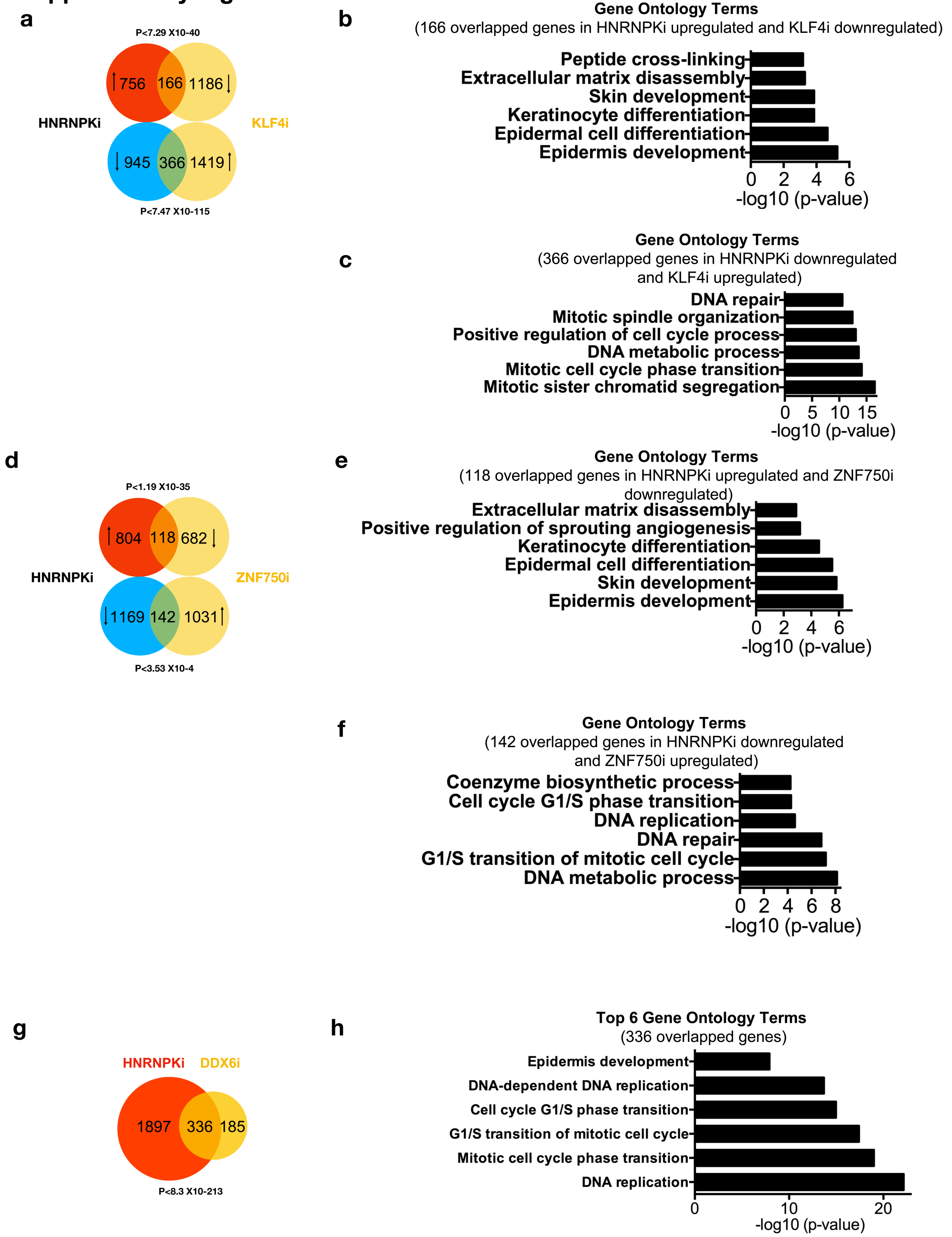

Supplementary Figure 3. Shared Gene Expression Signatures Between HNRNPK and Known Regulators of Epidermal Growth and Differentiation. Related to Figures 1-2.
(a) Venn diagram of overlapped genes between HNRNPKi and our previously published KLF4i gene expression profile. KLF4 knockdowns were performed in differentiation conditions while HNRNPK knockdown were performed in self-renewing proliferation

conditions. **(b)** Gene ontology (GO) terms of the 166 upregulated genes in HNRNPKi cells and downregulated genes in KLF4i cells. **(c)** Gene ontology terms of the 366 downregulated genes in HNRNPKi cells and upregulated genes in KLF4i cells. **(d)** Venn diagram of overlapped genes between HNRNPKi and our previously published ZNF750i gene expression profile. ZNF750 knockdowns were performed in differentiation conditions. **(e)** GO terms of the 118 upregulated genes in HNRNPKi cells and repressed genes in ZNF750i cells. **(f)** GO terms of the 142 repressed genes in HNRNPKi cells and upregulated genes in ZNF750i cells. **(g)** Venn diagram of overlapped genes between HNRNPKi and our previously published DDX6i gene expression profile. DDX6 and HNRNPK knockdowns were performed in self-renewing proliferation conditions. **(h)** GO terms of the 336 overlapped genes between DDX6i and HNRNPKi cells. Overlap significance in Venn diagrams was determined using hypergeometric distribution p-values (3a, 3d, 3g).

Supplementary Figure 4

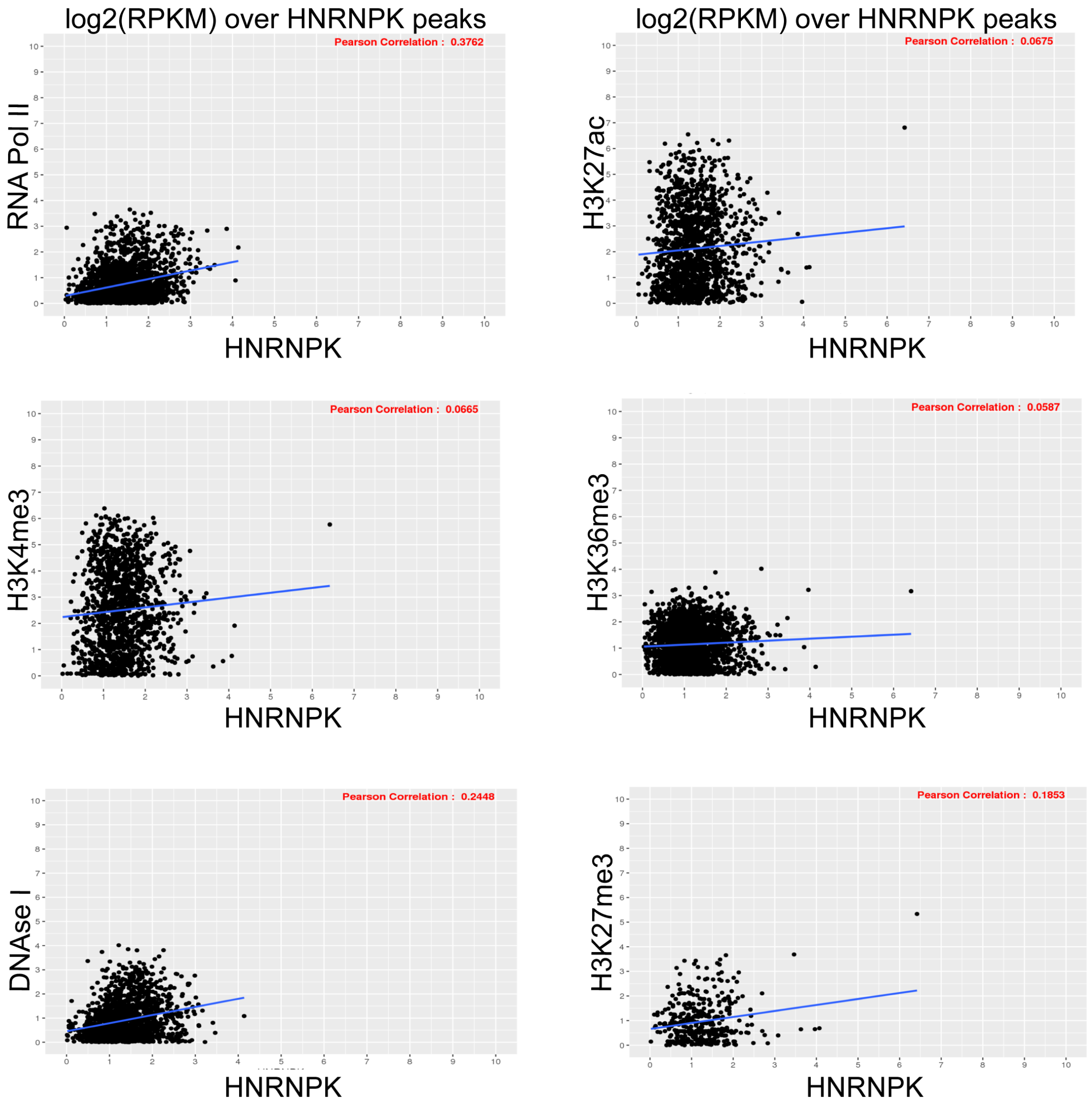

Supplementary Figure 4. HNRNPK ChIP-Seq correlates with RNA POL II binding sites. Supports Figure 4.
Comparison of log2 RPKM for HNRNPK and RNA Pol II, H3K27ac, H3K4me3, H3K36me3, DNAse I, and H3K27me3 ChIP-Seq signals at HNRNPK peak regions. Pearson correlation coefficient was calculated and regression line was plotted.

Supplementary Figure 5

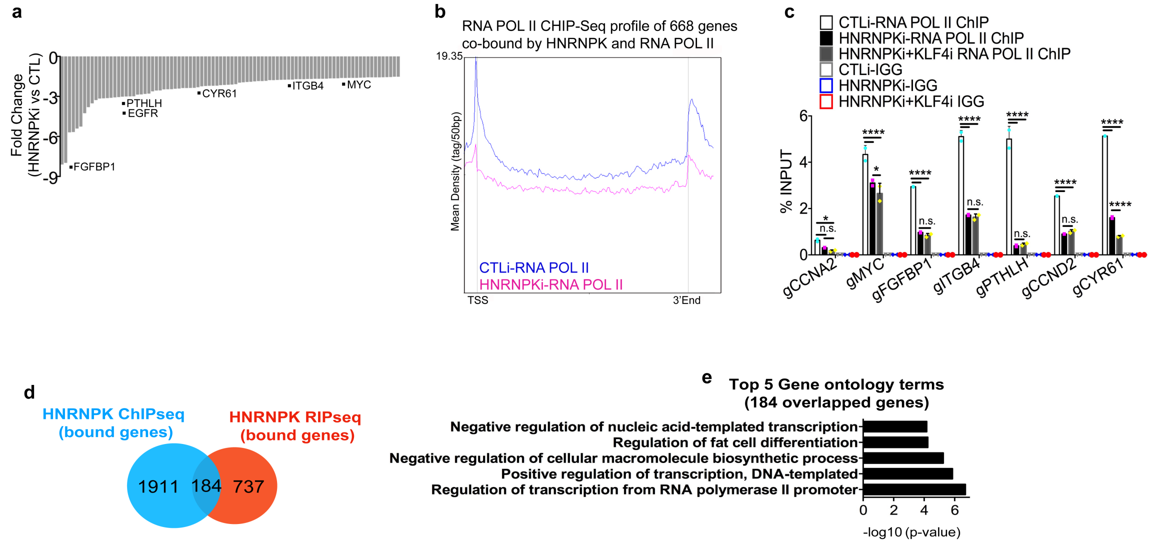

Supplementary Figure 5. RNA POL II loading onto HNRNPK co-bound genes is not due to the differentiation status of the cells. Supports Figure 6. (a) Genes co-bound by HNRNPK and RNA Pol II that lose RNA Pol II binding and are downregulated upon HNRNPK knockdown. **(b)** The distribution of RNA Pol II binding from the TSS to past the 3'end of genes of 668 genes co-bound by HNRNPK and RNA Pol II that don't significantly lose RNA Pol II binding HNRNPK knockdown. The purple track shows the RNA Pol II ChIP-Seq profiles across the 668 genes in control (CTL) cells. The pink track shows the RNA Pol II ChIP-Seq profiles across the 668 genes in HNRNPK knockdown cells. The y-axis denotes signal strength (mean density of the reads) and x-axis shows position from the TSS. **(c)** ChIP was performed on CTLi (white bar), HNRNPKi (black bar), and HNRNPKi+KLF4i (grey bar) cells using a RNA Pol II antibody. ChIP was also performed using IGG as a specificity control in CTLi (non filled light grey bar), HNRNPKi (non filled blue bar), and HNRNPKi+KLF4i (non filled red bar) cells. RNA Pol II binding to each gene was calculated as a percentage of input. N=2. Mean values are shown with error bars=SD. n.s.= not significant, *p <0.05, ****p <0.0001 (2 way ANOVA followed by Tukey's multiple comparison test). **(d)** Overlap of the 2,095 genes that HNRNPK binds on the DNA level (HNRNPK ChIP-Seq) with the 921 genes that HNRNPK binds on the mRNA level (HNRNPK RIP-Seq). **(e)** Gene ontology analysis of the 184 overlapped genes from (d).

Supplementary Figure 6

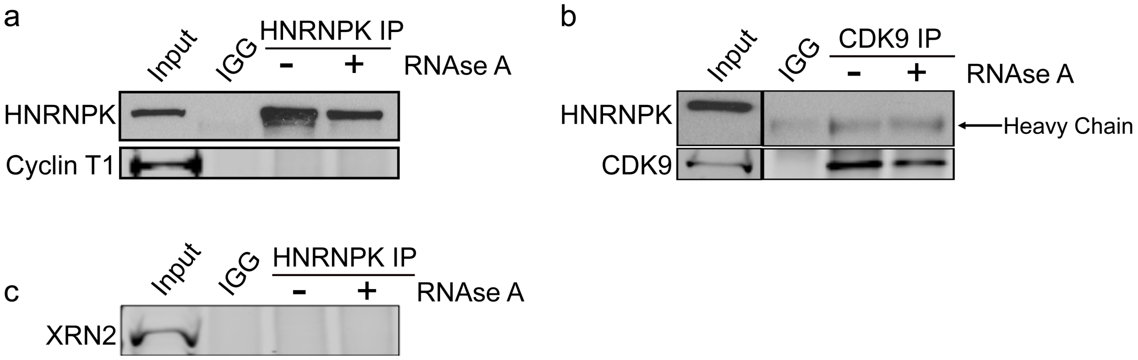

Supplementary Figure 6. HNRNPK does not associate with mediators of transcription elongation (CDK9, Cyclin T1) or termination (XRN2). Supports Figures 5-6.
Immunoprecipitations (IPs) were performed using either an HNRNPK, CDK9 antibody or IGG and Western blotted for HNRNPK, Cyclin T1, CDK9, or XRN2 protein expression. IPs were performed +/- RNase A. 5% of the cell lysate was used as input. Representative blots are shown, N=3.

Supplementary Figure 7

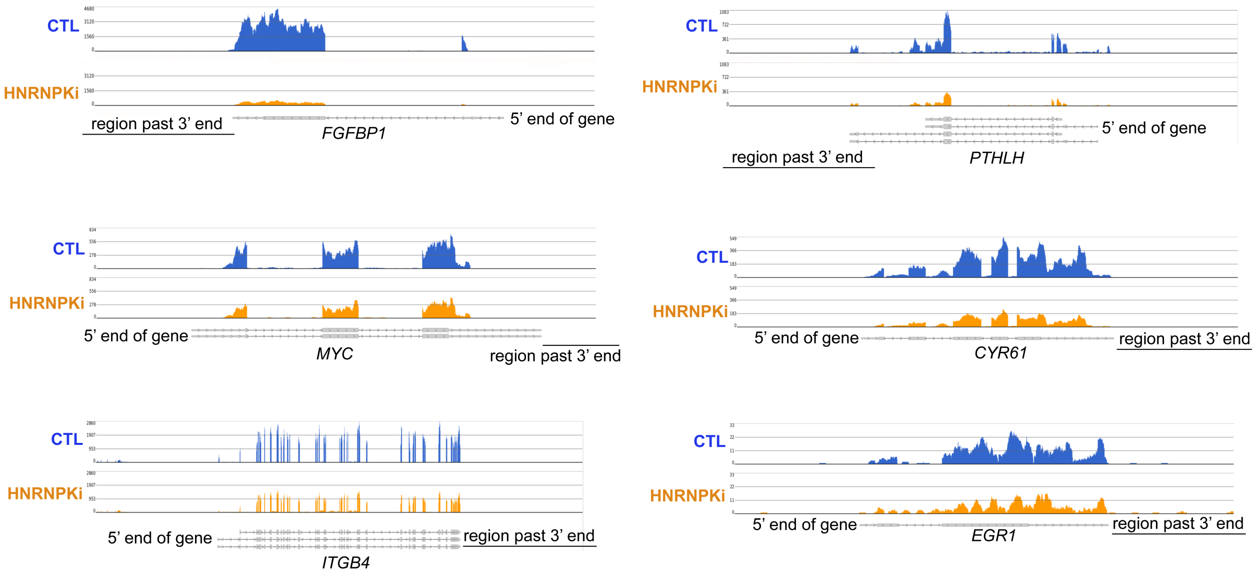

Supplementary Figure 7. HNRNPK does not impact transcription elongation or termination of proliferation genes. Supports Figures 5-7.
Gene tracks of Control (CTL) and HNRNPK knockdown (HNRNPKi) RNA-seq data on proliferation genes. The x-axis shows genomic position along each proliferation gene. The y-axis denotes signal strength (RPKM). N=2 for RNA-seq experiments for each sample.
