# Supplementary material for: HNRNPK maintains epidermal progenitor function through transcription of proliferation genes and degrading differentiation promoting mRNAs
Source: Nat Commun. 2019 Sep 13;10:4198. doi: 10.1038/s41467-019-12238-x (PMC6744489; doi:10.1038/s41467-019-12238-x)
Supplement: Supplementary file 2 — Reporting Summary [file 41467_2019_12238_MOESM2_ESM.pdf]

nature research

Corresponding author(s): George Sen
Last updated by author(s): Jul 24, 2019

Reporting Summary

Nature Research wishes to improve the reproducibility of the work that we publish. This form provides structure for consistency and transparency in reporting. For further information on Nature Research policies, see Authors & Referees and the Editorial Policy Checklist.

Statistics

For all statistical analyses, confirm that the following items are present in the figure legend, table legend, main text, or Methods section.

n/a	Confirmed	
☐	☒	The exact sample size (*n*) for each experimental group/condition, given as a discrete number and unit of measurement
☐	☒	A statement on whether measurements were taken from distinct samples or whether the same sample was measured repeatedly
☐	☒	The statistical test(s) used AND whether they are one- or two-sided<br>*Only common tests should be described solely by name; describe more complex techniques in the Methods section.*
☐	☒	A description of all covariates tested
☐	☒	A description of any assumptions or corrections, such as tests of normality and adjustment for multiple comparisons
☐	☒	A full description of the statistical parameters including central tendency (e.g. means) or other basic estimates (e.g. regression coefficient) AND variation (e.g. standard deviation) or associated estimates of uncertainty (e.g. confidence intervals)
☐	☒	For null hypothesis testing, the test statistic (e.g. *F*, *t*, *r*) with confidence intervals, effect sizes, degrees of freedom and *P* value noted<br>*Give P values as exact values whenever suitable.*
☒	☐	For Bayesian analysis, information on the choice of priors and Markov chain Monte Carlo settings
☒	☐	For hierarchical and complex designs, identification of the appropriate level for tests and full reporting of outcomes
☒	☐	Estimates of effect sizes (e.g. Cohen's *d*, Pearson's *r*), indicating how they were calculated

Our web collection on statistics for biologists contains articles on many of the points above.

Software and code

Policy information about availability of computer code

Data collection	*Provide a description of all commercial, open source and custom code used to collect the data in this study, specifying the version used OR state that no software was used.*
Data analysis	Bar plots were drawn using GraphPad Prism v7. Software used for RNA-Seq and ChIP-Seq analysis include: TopHat2 (Kim, D., Pertea, G., Trapnell, C., Pimentel, H., Kelley, R., and Salzberg, S.L. (2013). TopHat2: accurate alignment of transcriptomes in the presence of insertions, deletions and gene fusions. Genome biology 14), MACS2 (Zhang, Y., Liu, T., Meyer, C.A., Eeckhoute, J., Johnson, D.S., Bernstein, B.E., Nusbaum, C., Myers, R.M., Brown, M., Li, W., et al. (2008). Model-based analysis of ChIP-Seq (MACS). Genome biology 9))., seqMINER (Ye, T., Krebs, A.R., Choukrallah, M.A., Keime, C., Plewniak, F., Davidson, I., and Tora, L. (2011). seqMINER: an integrated ChIP-seq data interpretation platform. Nucleic Acids Res 39), Enrichr (Kuleshov, M.V., Jones, M.R., Rouillard, A.D., Fernandez, N.F., Duan, Q., Wang, Z., Koplev, S., Jenkins, S.L., Jagodnik, K.M., Lachmann, A., et al. (2016). Enrichr: a comprehensive gene set enrichment analysis web server 2016 update. Nucleic Acids Res 44), Partek Genomics Suite (http://www.partek.com/partek-genomics-suite/), Kundaje ChIP-seq pipeline (https://github.com/kundajelab/chipseq_pipeline)

For manuscripts utilizing custom algorithms or software that are central to the research but not yet described in published literature, software must be made available to editors/reviewers. We strongly encourage code deposition in a community repository (e.g. GitHub). See the Nature Research guidelines for submitting code & software for further information.

Data

Policy information about availability of data

All manuscripts must include a data availability statement. This statement should provide the following information, where applicable:

- Accession codes, unique identifiers, or web links for publicly available datasets
- A list of figures that have associated raw data
- A description of any restrictions on data availability

Provide your data availability statement here.

Field-specific reporting

Please select the one below that is the best fit for your research. If you are not sure, read the appropriate sections before making your selection.

☒ Life sciences ☐ Behavioural & social sciences ☐ Ecological, evolutionary & environmental sciences

For a reference copy of the document with all sections, see nature.com/documents/nr-reporting-summary-flat.pdf

Life sciences study design

All studies must disclose on these points even when the disclosure is negative.

Sample size	No sample-size calculations were performed. Sample size was determined to be adequate based on the magnitude and consistency of measurable differences between groups.
Data exclusions	Data were only excluded for failed experiments resulting from technical issues, such as uneven loading of gels, graft failures due to premature removal of bandages from grafted mice, etc..
Replication	Replicate experiments were successful.
Randomization	No randomization of mice. Mice analyzed were litter mates and sex-matched whenever possible.
Blinding	Investigators were not blinded to group allocation.

Reporting for specific materials, systems and methods

We require information from authors about some types of materials, experimental systems and methods used in many studies. Here, indicate whether each material, system or method listed is relevant to your study. If you are not sure if a list item applies to your research, read the appropriate section before selecting a response.

Materials & experimental systems

n/a	Involved in the study	
☐	☒	Antibodies
☐	☒	Eukaryotic cell lines
☐	☐	Palaeontology
☐	☒	Animals and other organisms
☐	☐	Human research participants
☐	☐	Clinical data

Methods

n/a	Involved in the study	
☐	☒	ChIP-seq
☐	☐	Flow cytometry
☐	☐	MRI-based neuroimaging

Antibodies

Antibodies used	HNRNPK (Bethyl Labs,A300-674A), Beta-Actin (Santa Cruz,sc47778), DDX6 (Novus Biologicals, NB200-192) RNA Pol II (Active Motif, 39097, clone 4H8), Keratin 1 (Biolegend, PRB-149P), Filaggrin (Abcam,Ab3137), MKI67 (Abcam, Ab16667), Keratin 10 (Abcam, Ab9025)
Validation	The HNRNPK antibody has been validated by the manufacturer (https://www.bethyl.com/product/A300-674A/hnRNP-K +Antibody) as well as referenced in 3 publications which can be found at the manufacturers website. We have also validated this antibody by in our current work by knockdown of HNRNPK and observing that the signal disappears on the Western blot and ChIP-QPCR level.

The DDX6 antibody has been validated by the manufacturer (https://www.novusbio.com/products/ddx6-antibody_nb200-192) as well as 16 publications referenced on the manufacturer's website. We have also thoroughly validated this antibody in our previous publication: Wang, Y., Arribas-Layton, M., Chen, Y., Lykke-Andersen, J. & Sen, G. L. DDX6 Orchestrates Mammalian Progenitor Function through the mRNA Degradation and Translation Pathways. Mol Cell 60, 118-130, doi:10.1016/j.molcel.2015.08.014 (2015). We have also validated this antibody in the current work by knocking down DDX6 and observing a diminished signal in Western blots.

The RNA Pol II has been thoroughly validated by the manufacturer for Western blot, ChIP, ChIP-Seq as well as the 29 publications referencing this antibody for use in the above stated applications. This information can be found at https://www.activemotif.com/catalog/details/39097/rna-pol-ii-antibody-mab.

The beta-actin antibody has been validated by the manufacturer for Western blotting and has also received 352 reviews. More information can be found at: https://www.scbt.com/scbt/product/beta-actin-antibody-c4.

The Keratin 1, Filaggrin, MKI67, and Keratin 10 antibodies are commonly used in immunofluorescence and has been cited in numerous publications as well as validated on the manufacturer's website:
Keratin 1: https://www.biolegend.com/en-ie/products/keratin-1-polyclonal-antibody-purified-10951
MKI67: https://www.abcam.com/ki67-antibody-sp6-ab16667.html
Keratin 10: https://www.abcam.com/cytokeratin-10-antibody-rkse60-ab9025.html

Eukaryotic cell lines

Policy information about cell lines

Cell line source(s)	Amphotropic Phoenix Cells
Authentication	These cells were used to produce virus so that we can infect primary human keratinocytes. The appropriate cell line was authenticated from the efficient retroviral transduction that we achieved in our keratinocytes (which is monitored by successful knockdown of a gene or successful selection using puromycin).
Mycoplasma contamination	The phoenix cells are routinely screened for mycoplasma contamination and were all negative for mycoplasma.
Commonly misidentified lines (See ICLAC register)	*Name any commonly misidentified cell lines used in the study and provide a rationale for their use.*

Palaeontology

Specimen provenance	*Provide provenance information for specimens and describe permits that were obtained for the work (including the name of the issuing authority, the date of issue, and any identifying information).*
Specimen deposition	*Indicate where the specimens have been deposited to permit free access by other researchers.*
Dating methods	*If new dates are provided, describe how they were obtained (e.g. collection, storage, sample pretreatment and measurement), where they were obtained (i.e. lab name), the calibration program and the protocol for quality assurance OR state that no new dates are provided.*

☐ Tick this box to confirm that the raw and calibrated dates are available in the paper or in Supplementary Information.

Animals and other organisms

Policy information about studies involving animals; ARRIVE guidelines recommended for reporting animal research

Laboratory animals	NOD SCID GAMMA mice (NOD.Cg-Prkdcscid Il2rgtm1Wjl/SzJ) Stock number: 005557 were purchased from Jackson Labs. Both males and females 8 weeks of age were used.
Wild animals	*Provide details on animals observed in or captured in the field; report species, sex and age where possible. Describe how animals were caught and transported and what happened to captive animals after the study (if killed, explain why and describe method; if released, say where and when) OR state that the study did not involve wild animals.*
Field-collected samples	*For laboratory work with field-collected samples, describe all relevant parameters such as housing, maintenance, temperature, photoperiod and end-of-experiment protocol OR state that the study did not involve samples collected from the field.*
Ethics oversight	UCSD's Institutional Animal Care and Use Committee (IACUC).

Note that full information on the approval of the study protocol must also be provided in the manuscript.

Human research participants

Policy information about studies involving human research participants

Population characteristics	*Describe the covariate-relevant population characteristics of the human research participants (e.g. age, gender, genotypic information, past and current diagnosis and treatment categories). If you filled out the behavioural & social sciences study design questions and have nothing to add here, write "See above."*
Recruitment	*Describe how participants were recruited. Outline any potential self-selection bias or other biases that may be present and how these are likely to impact results.*
Ethics oversight	*Identify the organization(s) that approved the study protocol.*

Note that full information on the approval of the study protocol must also be provided in the manuscript.

Clinical data

Policy information about clinical studies

All manuscripts should comply with the ICMJE guidelines for publication of clinical research and a completed CONSORT checklist must be included with all submissions.

Clinical trial registration	*Provide the trial registration number from ClinicalTrials.gov or an equivalent agency.*
Study protocol	*Note where the full trial protocol can be accessed OR if not available, explain why.*
Data collection	*Describe the settings and locales of data collection, noting the time periods of recruitment and data collection.*
Outcomes	*Describe how you pre-defined primary and secondary outcome measures and how you assessed these measures.*

ChIP-seq

Data deposition

☒ Confirm that both raw and final processed data have been deposited in a public database such as GEO.

☒ Confirm that you have deposited or provided access to graph files (e.g. BED files) for the called peaks.

Data access links<br>*May remain private before publication.*	All ChIP-seq files (raw data and BED files) have been deposited in the GEO database (accession GSE122327) as stated in the Methods section of the manuscript. The secure token to access these files is the following: efovocumtfkbxgf
Files in database submission	GSM3464087: HNRNPK_ChIPseq_rep_1<br>GSM3464088: HNRNPK_ChIPseq_rep_2<br>GSM3464089: HNRNPK_ChIPseq_rep_3<br>GSM3464090: Input_ChIPseq_rep_1<br>GSM3464091: CTLi_Input_Pol2CHIP<br>GSM3464092: HNRNPKi_Input_Pol2CHIP<br>GSM3464093: CTLi_RNAPol2_CHIPseq_rep_1<br>GSM3464094: CTLi_RNAPol2_CHIPseq_rep_2<br>GSM3464095: HNRNPKi_RNAPol2_CHIPseq_rep_1<br>GSM3464096: HNRNPKi_RNAPol2_CHIPseq_rep_2
Genome browser session<br>(e.g. UCSC)	http://genome.ucsc.edu/cgi-bin/hgTracks?db=hg19&hubUrl=http://oncogx.ucsd.edu/projects/HNRNPK/hub19.txt

Methodology

Replicates	Replicates: 3 replicates for HNRNPK ChIP, 2 replicates for RNA Pol2 ChIP in CTLi and HNRNPKi cells.
Sequencing depth	Between 27 and 55 million reads were retained per library following quality filtering (details provided in Methods).
Antibodies	The following antibodies were used: HNRNPK (Bethyl Laboratories: A300-674A), RNA Pol II (Active motif: 39097) , Rabbit IgG (Millipore: 12-370) and mouse IgG (Abcam: ab18413).
Peak calling parameters	see Methods, section "ChIP-qPCR and ChIP-seq"
Data quality	see Methods, section "ChIP-qPCR and ChIP-seq"
Software	see Methods, section "ChIP-qPCR and ChIP-seq"

Flow Cytometry

Plots

Confirm that:

☐ The axis labels state the marker and fluorochrome used (e.g. CD4-FITC).

☐ The axis scales are clearly visible. Include numbers along axes only for bottom left plot of group (a 'group' is an analysis of identical markers).

☐ All plots are contour plots with outliers or pseudocolor plots.

☐ A numerical value for number of cells or percentage (with statistics) is provided.

Methodology

Sample preparation	*Describe the sample preparation, detailing the biological source of the cells and any tissue processing steps used.*
Instrument	*Identify the instrument used for data collection, specifying make and model number.*
Software	*Describe the software used to collect and analyze the flow cytometry data. For custom code that has been deposited into a community repository, provide accession details.*
Cell population abundance	*Describe the abundance of the relevant cell populations within post-sort fractions, providing details on the purity of the samples and how it was determined.*
Gating strategy	*Describe the gating strategy used for all relevant experiments, specifying the preliminary FSC/SSC gates of the starting cell population, indicating where boundaries between "positive" and "negative" staining cell populations are defined.*

☐ Tick this box to confirm that a figure exemplifying the gating strategy is provided in the Supplementary Information.

Magnetic resonance imaging

Experimental design

Design type	*Indicate task or resting state; event-related or block design.*
Design specifications	*Specify the number of blocks, trials or experimental units per session and/or subject, and specify the length of each trial or block (if trials are blocked) and interval between trials.*
Behavioral performance measures	*State number and/or type of variables recorded (e.g. correct button press, response time) and what statistics were used to establish that the subjects were performing the task as expected (e.g. mean, range, and/or standard deviation across subjects).*

Acquisition

Imaging type(s)	*Specify: functional, structural, diffusion, perfusion.*
Field strength	*Specify in Tesla*
Sequence & imaging parameters	*Specify the pulse sequence type (gradient echo, spin echo, etc.), imaging type (EPI, spiral, etc.), field of view, matrix size, slice thickness, orientation and TE/TR/flip angle.*
Area of acquisition	*State whether a whole brain scan was used OR define the area of acquisition, describing how the region was determined.*

Diffusion MRI ☐ Used ☐ Not used

Preprocessing

Preprocessing software	*Provide detail on software version and revision number and on specific parameters (model/functions, brain extraction, segmentation, smoothing kernel size, etc.).*
Normalization	*If data were normalized/standardized, describe the approach(es): specify linear or non-linear and define image types used for transformation OR indicate that data were not normalized and explain rationale for lack of normalization.*
Normalization template	*Describe the template used for normalization/transformation, specifying subject space or group standardized space (e.g. original Talairach, MNI305, ICBM152) OR indicate that the data were not normalized.*
Noise and artifact removal	*Describe your procedure(s) for artifact and structured noise removal, specifying motion parameters, tissue signals and physiological signals (heart rate, respiration).*
Volume censoring	*Define your software and/or method and criteria for volume censoring, and state the extent of such censoring.*

Statistical modeling & inference

Model type and settings	*Specify type (mass univariate, multivariate, RSA, predictive, etc.) and describe essential details of the model at the first and second levels (e.g. fixed, random or mixed effects; drift or auto-correlation).*
Effect(s) tested	*Define precise effect in terms of the task or stimulus conditions instead of psychological concepts and indicate whether ANOVA or factorial designs were used.*

Specify type of analysis: ☐ Whole brain ☐ ROI-based ☐ Both

Statistic type for inference<br>(See Eklund et al. 2016)	*Specify voxel-wise or cluster-wise and report all relevant parameters for cluster-wise methods.*
Correction	*Describe the type of correction and how it is obtained for multiple comparisons (e.g. FWE, FDR, permutation or Monte Carlo).*

Models & analysis

n/a	Involved in the study
☐	☐ Functional and/or effective connectivity
☐	☐ Graph analysis
☐	☐ Multivariate modeling or predictive analysis

Functional and/or effective connectivity	*Report the measures of dependence used and the model details (e.g. Pearson correlation, partial correlation, mutual information).*
Graph analysis	*Report the dependent variable and connectivity measure, specifying weighted graph or binarized graph, subject- or group-level, and the global and/or node summaries used (e.g. clustering coefficient, efficiency, etc.).*
Multivariate modeling and predictive analysis	*Specify independent variables, features extraction and dimension reduction, model, training and evaluation metrics.*
