# Supplementary material for: HNRNPK maintains epidermal progenitor function through transcription of proliferation genes and degrading differentiation promoting mRNAs
Source: Nat Commun. 2019 Sep 13;10:4198. doi: 10.1038/s41467-019-12238-x (PMC6744489; doi:10.1038/s41467-019-12238-x)
Supplement: Supplementary file 3 — Description of Additional Supplementary Files [file 41467_2019_12238_MOESM3_ESM.pdf]

Description of Additional Supplementary Files

Supplementary Data 1. Global Gene Expression Profiling Comparing Control and HNRNPKi Cells. Related to Figure 1.
RNA-Seq comparing control and HNRNPK knockdown cells. Samples were performed in biological duplicates. Significant changes between control (CTL) and HNRNPKi cells were identified by ANOVA with a p-value of less than or equal to 0.05 and an average fold change of greater than or equal to 2. Positive fold change values indicate fold increase in HNRNPK knockdown as compared to control (CTL). Negative fold change values indicate fold decrease in HNRNPK knockdown as compared to control (CTL)

Supplementary Data 2. HNRNPK RNA IP followed by next generation sequencing (RIP-Seq) Related to Figure 2.
HNRNPK or IGG RNA IP followed by high-thoughput sequencing on epidermal progenitor cells. Samples were performed in biological duplicates. Significant changes between HNRNPK and IGG were identified by ANOVA with a p-value of less than or equal to 0.05 and an average fold change of greater than or equal to 4 in HNRNPK pulldowns as compared to IGG.

Supplementary Data 3a. HNRNPK binding across the genome. Related to Figure 3.
HNRNPK ChIP-Seq showing the start and end of each binding site. The binding sites are also mapped back to its nearest gene.

Supplementary Data 3b. RNA POL II binding in control keratinocytes across the genome. Related to Figure 4.
RNA Pol II ChIP-Seq showing the start and end of each binding site. The binding sites are also mapped back to its nearest gene.

Supplementary Data 4. Differential binding of RNA POL II in CTLi and HNRNPKi cells across the genome. Related to Figure 5.
The peaks that show differential RNA Pol II binding upon HNRNPK knockdown are shown. These include peaks that are lost (down events) as well as gained (up events). Differentially identified peaks were called by DiffReps. The start and end of each differentially called region is shown as well as being mapped back to its nearest gene.
